# A methylation- and immune-related lncRNA signature to predict ovarian cancer outcome and uncover mechanisms of chemoresistance

**DOI:** 10.1186/s13048-023-01260-9

**Published:** 2023-09-06

**Authors:** Lu Chen, Wujiang Gao, Li Lin, Chunli Sha, Taoqiong Li, Qi Chen, Hong Wei, Meiling Yang, Jie Xing, Mengxue Zhang, Shijie Zhao, Wenlin Xu, Yuefeng Li, Lulu Long, Xiaolan Zhu

**Affiliations:** 1 Reproductive Medicine Center, The Fourth Affiliated Hospital of Jiangsu University, No. 20, Zhengdong Road, Zhenjiang City, 212001 Jiangsu Province China; 2 Department of Gynaecology and Obstetrics, Taixing People’s Hospital, Taixing, Jiangsu China; 3 Department of Gynaecology and Obstetrics, Yangzhou First People’s Hospital, Yangzhou, Jiangsu China; 4 Department of Gynaecology and Obstetrics, The First People’s Hospital of Nantong City, Nantong, Jiangsu China; 5 Medical school, Jiangsu University, No. 301, Xuefu Road, Zhenjiang City, 212031 Jiangsu Province China; 6 Oncology Department, Affiliated People’s Hospital of jiangsu university, No. 8, Dianli Road, Zhenjiang City, 212001 Jiangsu Province China

**Keywords:** mrlncRNA, irlncRNA, OC, Chemoresistance, Tumor-infiltrating

## Abstract

Tumor-associated lncRNAs regulated by epigenetic modification switches mediate immune escape and chemoresistance in ovarian cancer (OC). However, the underlying mechanisms and concrete targets have not been systematically elucidated. Here, we discovered that methylation modifications played a significant role in regulating immune cell infiltration and sensitizing OC to chemotherapy by modulating immune-related lncRNAs (irlncRNAs), which represent tumor immune status. Through deep analysis of the TCGA database, a prognostic risk model incorporating four methylation-related lncRNAs (mrlncRNAs) and irlncRNAs was constructed. Twenty-one mrlncRNA/irlncRNA pairs were identified that were significantly related to the overall survival (OS) of OC patients. Subsequently, we selected four lncRNAs to construct a risk signature predictive of OS and indicative of OC immune infiltration, and verified the robustness of the risk signature in an internal validation set. The risk score was an independent prognostic factor for OC prognosis, which was demonstrated via multifactorial Cox regression analysis and nomogram. Moreover, risk scores were negatively related to the expression of CD274, CTLA4, ICOS, LAG3, PDCD1, and PDCD1LG2 and negatively correlated with CD8^+^, CD4^+^, and Treg tumor-infiltrating immune cells. In addition, a high-risk score was associated with a higher IC50 value for cisplatin, which was associated with a significantly worse clinical outcome. Next, a competing endogenous RNA (ceRNA) network and a signaling pathway controlling the infiltration of CD8^+^ T cells were explored based on the lncRNA model, which suggested a potential therapeutic target for immunotherapy. Overall, this study constructed a prognostic model by pairing mrlncRNAs and irlncRNAs and revealed the critical role of the FTO/RP5-991G20.1/hsa-miR-1976/MEIS1 signaling pathway in regulating immune function and enhancing anticancer therapy.

**Supplementary Information:**

The online version contains supplementary material available at 10.1186/s13048-023-01260-9.

## Background

Ovarian cancer (OC) is the second leading cause of gynecological cancer death worldwide [1]. Due to the lack of timely diagnosis at an early stage and the emergence of chemoresistance at a late stage, most patients with OC have a poor prognosis [2, 3]. Therefore, distinguishing patients according to their treatment response and identifying the underlying mechanisms limiting the anticancer efficacy of chemotherapy drugs are crucial to ameliorate patient outcomes.

Recently, as the most promising treatment against cancers, immunotherapy has gradually revolutionized cancer treatment with the progression of research on tumor-immune interactions [4]. An increasing number of studies have demonstrated that the immune system plays a significant role in cancer initiation, progression, and therapeutic responses [5, 6]. Moreover, OC is an immunogenic tumor, the course of which can effectively be transformed by immunotherapy [7]. Previous researches reported the prognostic value of the OC immune system and revealed the importance of tumor-related signaling pathways in the tumor immune microenvironment [8]. Although several types of tumors exhibit effective responses to immunotherapy, especially immune checkpoint blockade, many patients fail to benefit from immunotherapy, which is related to the prognosis of various cancer types, and OC patients have a poor response to PD1/PD-L1 monotherapy [9]. Therefore, screening out these patients and providing therapeutic targets to improve patient prognosis are of great significance. In addition, a clinical situation has emerged in which the tumor recurred despite seemingly lasting remission at initial treatment [10, 11]. One of the possible explanations is an extra disruption of cancer-immune homeostasis. Consistent with this, mounting evidence has shown that the tumor immune microenvironment and systemic immune system strongly influence the efficacy of anticancer drugs [12]. Local tumor immunity and systemic immunity can enhance or weaken the effect of anticancer treatment by regulating the composition and characteristics of the tumor microenvironment [13]. This reminds us of the great significance of determining the mechanisms of immunity regulation in resensitizing patients with OC to anticancer therapy. As reported, high immune cell infiltration (ICI)-scoring OC patients with better clinical overall survival (OS), higher tumor mutation burden (TMB), higher immune checkpoint expression (PD1, PD-L1, PD-L2 and CTLA4) and higher sensitivity to two first-line chemotherapy drugs (paclitaxel and cisplatin) might benefit from immunotherapy, which means that the ICI score is an effective prognosis-related biomarker of OC and can provide valuable information on the potential response to immunotherapy [14].

Interestingly, studies have revealed that long noncoding RNA (lncRNA) expression is reliably related to cancer prognosis. Additionally, lncRNAs involved in the infiltration, differentiation and function of immune cells, have great predictive value for the immune response [15, 16]. And lncRNAs, play a key role in a wide range of biological processes, including the regulatory network of regulator gene, which plays a key role in cancer progression. Notably, an increasing number of clinical studies have also shown potential in diagnosis, prediction of prognosis, and therapeutic targets for OC [17, 18]. However, only limited comprehensive investigations focused on the molecular regulatory mechanism between lncRNAs and immune infiltration have been conducted [19]. Given the above factors, lncRNAs would be an excellent choice as immune biomarkers to predict OC prognosis. Furthermore, studies have indicated that lncRNAs are essential regulators in gene expression networks and participate in almost all biological processes, including tumorigenesis and progression, through diverse mechanisms at the transcriptional, posttranscriptional, and epigenetic levels [20, 21]. As documented, a risk model based on 4 lncRNAs (CACNA1G-AS1, ACAP2-IT1, AC010894.3 and UBA6-AS1) involved in m6A regulation was identified to predict OS and therapeutic value in OC independently [22].

Methylation modification acts as an essential component of epigenetic modifications associated with multiple pathological processes [23]. Recent studies have presented methylation modification, which exists in mRNAs and lncRNAs, as an emerging mechanism in gene regulation [24, 25]. To date, the six most prominent types of methylation modifications in the human genome have been reported, comprising N1-methyladenosine (m1A), N6-methyladenosine (m6A), 5-methylcytosine (m5C), N7-methylguanosine (m7G), N6,2'-O-dimethyladenosine (m6Am), and 5-hydroxymethylcytosine (hm5C) [26]. This reversible posttranscriptional modification is regulated by methylation regulators, which are usually classified as “writers”, “erasers”, and “readers” based on their functions. The “writers” (methyltransferases) catalyze methylation. The “erasers” (demethylase), remove methylation modifications from RNA. Readers, as methylation binding proteins, recognize methylation and generate functional signals SSSS [27]. Increasing evidence has demonstrated that methylation modifications participate in the progression of cancers, such as glioma, breast cancers, hepatocellular carcinoma and ovarian cancer [28–30]. In terms of the importance of methylation and immune infiltration in tumors, it is conceivable that immune status alterations caused by the methylation modification of lncRNAs could contribute to cancer treatment. Additionally, the deregulation of lncRNAs by epigenetic alterations has been implicated in cancer initiation and progression. For example, the lncRNA SNHG12 acts as a mediator of chemoresistance in OC via epigenetic mechanisms [31], and the N-methyladenosine reader YTHDF2 mediates lncRNA FENDRR degradation, promoting endometrial cancer (EC) progression [32].

Moreover, studies have indicated that m6A modification of lncRNA MIR155HG promotes immune escape of hepatocellular carcinoma cells to upregulate PD-L1 expression [33]. Similarly, the methylation level of lncRNA FAM83H-AS1 was related to the gene expression of FAM83H-AS1, which was related to immune cell infiltration in OC patients [34]. These findings further inspired us to conclude that methylation modification of lncRNAs can modulate the immune microenvironment and thus modulate tumor cell biology characteristics. However, a systematic analysis is still lacking. Therefore, we hypothesized that methylation modification could affect the immune status and prognosis of OC by regulating immune-related lncRNAs. Here, we aimed to not only provide a promising immune prognostic model for predicting outcome and immune response but also explore the regulatory mechanism of tumor immunity conditions, thus helping to overcome the obstacles in immune evasion and chemoresistance of OC.

## Methods

### Clinical and profiling data

Clinical and profiling data were downloaded from The Cancer Genome Atlas (TCGA) database (https://portal.gdc.cancer.gov/cart; up to January 9, 2022). The inclusion criteria were as follows: (1) the primary disease was diagnosed as ovarian cancer, removing patients who have ever been affected by other malignant tumors; (2) Patients with complete follow-up information including survival time, survival status, outcome, age, sex, clinical stage, and grade were selected to match with their RNA seq data. The main outcome of our study was overall survival. Patients without survival information were removed for further evaluation. Following these criteria, 363 OC cases were included. Besides, we have provided more details about clinical information of OC cases in Supplementary Table 1. The transcription data were scale normalized.

### Methylation-related genes

The methylation-related genes are 50 recognized methylation regulators extracted from previous studies including “writers”, “erasers”, and “readers” [28, 35–37]. We obtained the profiles of methylation-related genes from the TCGA database. the expression matrixes of 50 methylation-related genes were retrieved from TCGA, including the expression data of 32 writers, 14 readers and 7 erasers (Supplementary Table 2).

### Immune-related genes

We obtained immune-related genes of OC from previous study [38]. In this study, the authors divided the 308 ovarian cancer samples from TCGA into high- and low-abundance immune subtypes based on the abundance of immune cell infiltration. Differentially Expressed Genes (DEG) between these two groups were determined with the R package limma package (Bioconductor version 3.0). Meanwhile, the authors downloaded immune-related genes from the website (IMMPORT: https://www.immport.org/) in which genes with different immune functions were included. Then, the intersection of the DEGs and immune-related genes were obtained, which include 98 differentially expressed immune-related genes of OC. In our study, we used these 98 immune-related genes to obtain immune-related lncRNA for follow-up research (Supplementary Table 3).

### Selection of immune- and methylation-related lncRNAs

Based on the level 3 data provided in TCGA, according to the classification of gene type in the Ensembl (http://www.ensembl.org/) database, non-coding protein genes were selected as ncRNAs, and those with lengths greater than 200 bp were selected as lncRNAs. The expression matrixes of 50 methylation-related genes and 98 immune-related genes were retrieved from TCGA.

Methylation gene-related lncRNAs and immune gene-related lncRNAs by Pearson’s correlation analysis were calculated in 363 samples. Then, the 789 immune-related lncRNAs were identified for high correlation with the immune score (|Pearson R|> 0.35&P < 0.05) as several published articles have constructed several immune related lncRNA models [39, 40]. After screening methylation gene-related lncRNAs and immune gene-related lncRNAs by Pearson’s correlation analysis, 1688 methylation-related lncRNAs (mrlncRNAs) and 789 immune-related lncRNAs (irlncRNAs) were identified. The criteria of |Pearson R|> 0.35 and p < 0.05 were used in the process. We selected 326 lncRNAs of the intersection of methylation-related lncRNAs (mrlncRNAs) and immune-related lncRNAs.

Before construction of a prognostic model, 21 lncRNAs were screened out significantly correlated with OS (p < 0.05) from the 326 lncRNAs. Then, we used computer-generated random numbers to assigned the 363 TCGA-OV samples into a row and numbered them 1–363 randomly, took a 50% random number of 182 patients as the Training group, and the remaining 181 patients as the Validation group. The training set was utilized to construct mrlncRNA and irlncRNA risk models. Some published articles have also constructed and validated relevant risk prediction models based on this way [37, 40].

### Survival analysis

Univariate Cox analysis was implemented by the survival package to investigate the prognostic value of lncRNAs in TCGA-OV patients (https://mirrors.tuna.tsinghua.edu.cn/CRAN/web/ packages/survival/index.html). Only lncRNAs with a *P* value < 0.05 were considered to be significantly associated with survival. The coxpbc, nomogram, cph, calibrate, and cindex functions in R were used to construct, validate, and calibrate the nomogram.

### Construction and validation of an immune-related prognostic model

To construct the prognostic model, TCGA ovarian cancer samples were randomly divided into a training set and a validation set at a ratio of 1:1. In the training set, lncRNAs related to prognosis were screened by univariate Cox regression, and then the model was simplified by least absolute shrinkage and selection algorithm (LASSO) regression analysis (tenfold cross validation). Finally, the optimal model was obtained by Cox proportional risk regression analysis. The model followed the Akaike information criterion (AIC), and the model with the lowest AIC value was selected as the optimal model, The survminer package of R software was used to analyze the best cutoff value of this model and group the training set (the number of samples in the group was greater than 30% of the total number of samples in the training set). The log⁃rank test was used to compare the differences in TCGA-OV in the training set and validation set and generate the survival curve; The receiver operating characteristic (ROC) curves of 1, 3 and 5 years were analyzed. The discrimination of the model was evaluated by the area under the curve (AUC) and the C⁃index. The survival of the training set and validation set were analyzed with the same cutoff value. Then, the relationship among age, clinical-stage, grade, risk-group and the prognosis of ovarian cancer was analyzed by univariate and multivariate Cox regression in the training set and validation set. Finally, the 1-, 3- and 5-year survival rates were predicted by constructing a nomogram, and evaluated by a calibration curve (followed by the bootstrap method). The calibration curve showed the fitting degree between the predicted survival rate and the actual survival rate of the nomogram, to evaluate the prediction accuracy of the nomogram. Finally, the benefit of the nomogram in guiding clinical decision-making was evaluated by 5-year decision-making curve analysis, and compared with other pathological parameters. The above analysis process was completed by R4.0.3 (http://www.r-project.org) and SPSS 21.0 software. Unless otherwise specified, two-tailed *P* < 0.05 was considered to indicate statistical significance.

### Estimation of immune cell type fractions

As a method based on gene expression profiles to characterize the cell composition of complex tissues, CIBERSORT is highly consistent with the basic truth estimations in many cancers. LM22, as a leukocyte gene signature matrix consisting of 547 genes, was used to distinguish 22 immune cell types including myeloid subsets, natural killer (NK) cells, plasma cells, naive and memory B cells and seven T cell types. CIBERSORT was combined with the LM22 signature matrix to estimate the fractions of these 22 immune cell types between the risk score and immune infiltration degree or immune checkpoints. The sum of all estimated immune cell type fractions is equal to 1 for each sample.

### Construction or identification of markers associated with chemoresistance

The pRRophetic package was used to construct or identify markers associated with chemoresistance. Additionally, it was used to analyze the relationship between prognostic models and immune infiltration, checkpoint expression, drug resistance and IC50 of cisplatin.

### Construction of ceRNA networks

The GDCRNATools package in R software was used to establish a ceRNA network. The interactive relations in both lncRNA-miRNA and miRNA-mRNA were derived from StarBase (http://mirtarbase.mbc.nctu.edu.tw/). The miRNAs with significant outcomes in hypergeometric testing and correlation analysis were singled out to construct ceRNA networks. Then, ceRNA networks centered on mrlncRNAs and irlncRNAs were constructed by employing the miRanda algorithm (http://www.microrna.org/) and the PITA algorithm (https://genie.weizmann.ac.il/pubs/mir07/mir07_exe.html). Ultimately, Cytoscape version 3.4.0 was used to visualize ceRNA networks.

### Cell culture and treatment

Human ovarian cancer cell line A2780 was obtained from Shanghai Institute of Cell Biology,China Academy of Sciences.Cells were cultured in RPMI medium modified supplemented with 10% fetal calf serum (Gibco BRL, Grand Island, NY), 100 U/mL penicillin and 100 µg/mL streptomycin in a humidified incubator containing 5% CO2 at 37 °C.

### Tissue samples

With the approval of the Jiangsu University ethics committee, epithelial ovarian cancer samples from patients with FIGO stage IIIC or IV were collected at Zhenjiang Maternal and Child Health Hospital (The Fourth Affiliated Hospital of Jiangsu University) and The Affiliated People's Hospital of Jiangsu University. All patients were treated with the standard care of platinum-based therapy after surgery, and informed consent was obtained from all patients. PFS was calculated from the time of surgery to the time of progression or recurrence. Platinum resistance or platinum sensitivity was defined as a relapse or progression within 6 months or 6 months after the last platinum-based chemotherapy, respectively. Each group had more than 12 patient samples. Clinical and pathological features are described in Table 1.

**Table 1:** Clinical and pathological features of EOC patients

Characteristics	Age (years)	Pathological type	FIGO stage	Grade	progression-free survival (months)
EOC1	66	serous mucinous carcinoma	IIIA1(i)	Low	> 6
EOC2	62	serous carcinoma	IIIA2	Moderate	> 6
EOC3	70	mucinous carcinoma	IVA	High	> 6
EOC4	65	serous carcinoma	IIIC	High	> 6
EOC5	63	serous mucinous carcinoma	IIIC	Moderate	> 6
EOC6	64	serous carcinoma	IVA	Moderate	> 6
EOC7	68	serous carcinoma	IIIC	High	> 6
EOC8	67	clear cell carcinoma	IVA	High	> 6
EOC9	69	endometrioid carcinoma	IIIA1(ii)	Low	> 6
EOC10	71	mucinous carcinoma	IIIC	Moderate	> 6
EOC11	65	serous carcinoma	IVA	Low	> 6
EOC12	59	serous carcinoma	IVA	High	> 6
EOC13	60	serous carcinoma	IIIC	High	< 6
EOC14	68	serous mucinous carcinoma	IIIC	Low	< 6
EOC15	68	mucinous carcinoma	IVA	High	< 6
EOC16	65	clear cell carcinoma	IIIC	High	< 6
EOC17	66	serous carcinoma	IIIB	Moderate	< 6
EOC18	69	serous carcinoma	IIIA2	Moderate	< 6
EOC19	72	serous carcinoma	IIIC	High	< 6
EOC20	66	serous carcinoma	IIIB	High	< 6
EOC21	70	serous carcinoma	IVB	High	< 6
EOC22	65	serous carcinoma	IIIC	Moderate	< 6
EOC23	76	serous carcinoma	IIIC	High	< 6
EOC24	60	serous carcinoma	IVA	High	< 6

### siRNA

The si-FTO and its corresponding negative control siRNA were purchased from Suzhou GenePharma Co.,Ltd (Suzhou, China). The details on siRNA are described in Table 2.

**Table 2:** Details on siRNA

Plasmids	Source	Species	Sense(5’-3’)	Anti-sense(5’-3’)
si-FTO	GenePharma Co.,Ltd	Human	UCGCAUCCUCAUUGGUAAUTT	AUUACCAAUGAGGAUGCGAGA

### Cisplatin

Cisplatin(cDDP) is provided by the central pharmacy of the Fourth People's Hospital of Zhenjiang City, and its diluent is phosphate-buffered saline (PBS). The details on siRNA are described in Table 3.

**Table 3:** Details on Cisplatin

Drug	Source	Approval number	Specification	Diluent	Concentration (μg/ml)
Cisplatin	QiLu Pharmaceutical (Hainan) Co., Ltd	H20073652	10 mg	phosphate-buffered saline (PBS)	1, 2, 4, 8, 16, 32

### Transfection

Cells were seeded into 6-well plates (6 × 10^5^ cells/well) and transfected with si-FTO and its negative control (Suzhou GenePharma Co.,Ltd, Suzhou, China) at a final concentration of 100 nM using Lipofectamine 2000 which concentration was 4 μl per well (Invitrogen, CA, USA). The time course is as follows: 1) 1.5 ml basic culture medium (serum-free and triple antibody free) was used to starve experimental cells for 1 h; 2) 250 μl of basic culture medium was mixed with 1 μl of si-FTO and its negative control; 3) 250 μl of basic culture medium was mixed with 4 μl of Lipofectamine 2000; 4) Fully mixed 2) and 3); 5) After incubating in incubator for 4–6 h, replaced with a complete medium (including serum and triple antibody) and incubated for another 24 h; 6) Collect samples and extract RNA.

### Colony forming assay

The cells were plated in 6-well dishes (500 cells/well) and exposed to a specific dose of cDDP(0.5 µg/ml), and subsequently grown for 14 days. Next, the cells were fixed with 4% Paraformaldehyde (PFA), which were then stained with 0.3% crystal violet. Colonies containing more than 50 cells were identified using densitometry software (Image J) and scored as survivors.

### Evaluation of proliferation and apoptosis

To evaluate cell proliferation and apoptosis, 6 × 10^5^ cells were seeded into 6-well plates. After transfection, EOC cells were treated with cDDP and PPAR inhibitor for 24 h. Terminal deoxynucleotidyl transferase dUTP nick end labeling (TUNEL) (In situ cell death detection kit, Fluorescein, Roche, Basel, Switzerland) was used to assess apoptosis according to the instructions. Also, the proliferative ability of ECO cells were analyzed by the the EDU (5-Ethynyl -2’- deoxyuridine) cell proliferation test kit(Ribobio, Guangzhou, China). The weaker the proliferation signal, the stronger the apoptotic ability of the tested cells, and vice versa.

### Assessment of chemosensitivity to cDDP

The control group and FTO knockdown cells were separately plated into 96-well plates (5 × 10^3^ cells/well) and exposed to various doses of cDDP (1, 2, 4, 8, 16 and 32 µg/ml). Then, 10 μl of CCK-8 solution (Vazyme, Nanjing, China) was added to each well, and the plate was incubated for 2 h in a humidified incubator. The absorbance of each well was measured at 450 nm using a Model 550 series microplate reader (Bio-Rad Laboratories). Cell viability was expressed as the ratio of treated cells to untreated controls at each dose or concentration. The IC50 value for each cell line was determined by nonlinear regression analysis using GraphPad Prism (GraphPad Software Inc., San Diego, CA).

### Real-time Quantitative PCR

Total RNA was isolated using Trizol reagent. cDNA as synthesized using a FastQuant RT Kit (with gDNase) (#KR106, Tiangen, Shanghai, China) according to the manufacturer’s instructions. Quantitation of RNAs was carried out using a miRcute Plus RNA qPCR Detection Kit (#FP411, Tiangen). The raw qRT-PCR RNAs data were normalized to the spiked GAPDH or U6 levels as described previously. The quantitative PCR procedures were carried out with real-time PCR SYBR Green q-PCR Super-mix. The RNA expression levels were analyzed and quantified by calculating using the 2 − ^ΔΔ^Ct method. The PCR cycle parameters are as follows: 1) Pre denaturation at 95℃ for 15 min; 2) 95℃ denaturation for 10 s; 3) Annealing at 48℃ for 20 s; 4) 72℃ extension for 30 s; 5) a total of 45 cycles. The primers of RNA are listed in Table 4. The primer concentrations were provided in Table 5.

**Table 4:** Primers used for qRT-PCR

PRIMER	FORWARD(5’-3’)	REVERSE(5’-3’)
FTO	ACTTGGCTCCCTTATCTGACC	TGTGCAGTGTGAGAAAGGCTT
RP5-991G20.1	TGTTGCTCTTCTTCATGGCTCGTG	AGTGGATGGCTTCAATCTCGGTATG
hsa-miR-1976	CTCCTGCCCTCCTTGCTG	-
MEIS1	CTTCCCTCTCTTAGCACTGATT	AAAATAGAGGTTTTTCTGCGCG
GAPDH	AATGCATCCTGCACCACCAA	GTAGCCATATTCATTGTCATA
U6	CTCGCTTCGGCAGCACA	AACGCTTCACGAATTTGCGT

**Table 5:** The qRT-PCR reaction system

REAGENT	VOLUME
2xSuperReal Color PreMix	5 μl
Forward(10 μM)	0.3 μl
Reverse(10 μM)	0.3 μl
cDNA templet	2 μl
Rnase Free ddH2O	Fill in to 10 μl

### Statistical analysis

All numeric data are presented as the means ± standard deviations (SDs) of at least three independent experiments. The experimental results were analyzed by analysis of variance or two-tailed Student’s t test at a significance level of *P* < 0.05 (*P < 0.05, **P < 0.01 and ***P < 0.001) using Prism 5 software (GraphPad Software, San Diego, CA, USA). A *P* value < 0.05 was considered to indicate statistical significance. The remaining statistical analysis were performed with R version 4.0.3 software (package: GDCRNATools DEseq2, edgeR, ggplot2, clusterProfiler, glmnet, preprocessCore, survminer, survival, timeROC, rms, pheatmap, corrplot, and vioplot).

## Results

### Development and validation process of the prognostic risk model and the strategy and workflow of the study

**Fig. 1:**
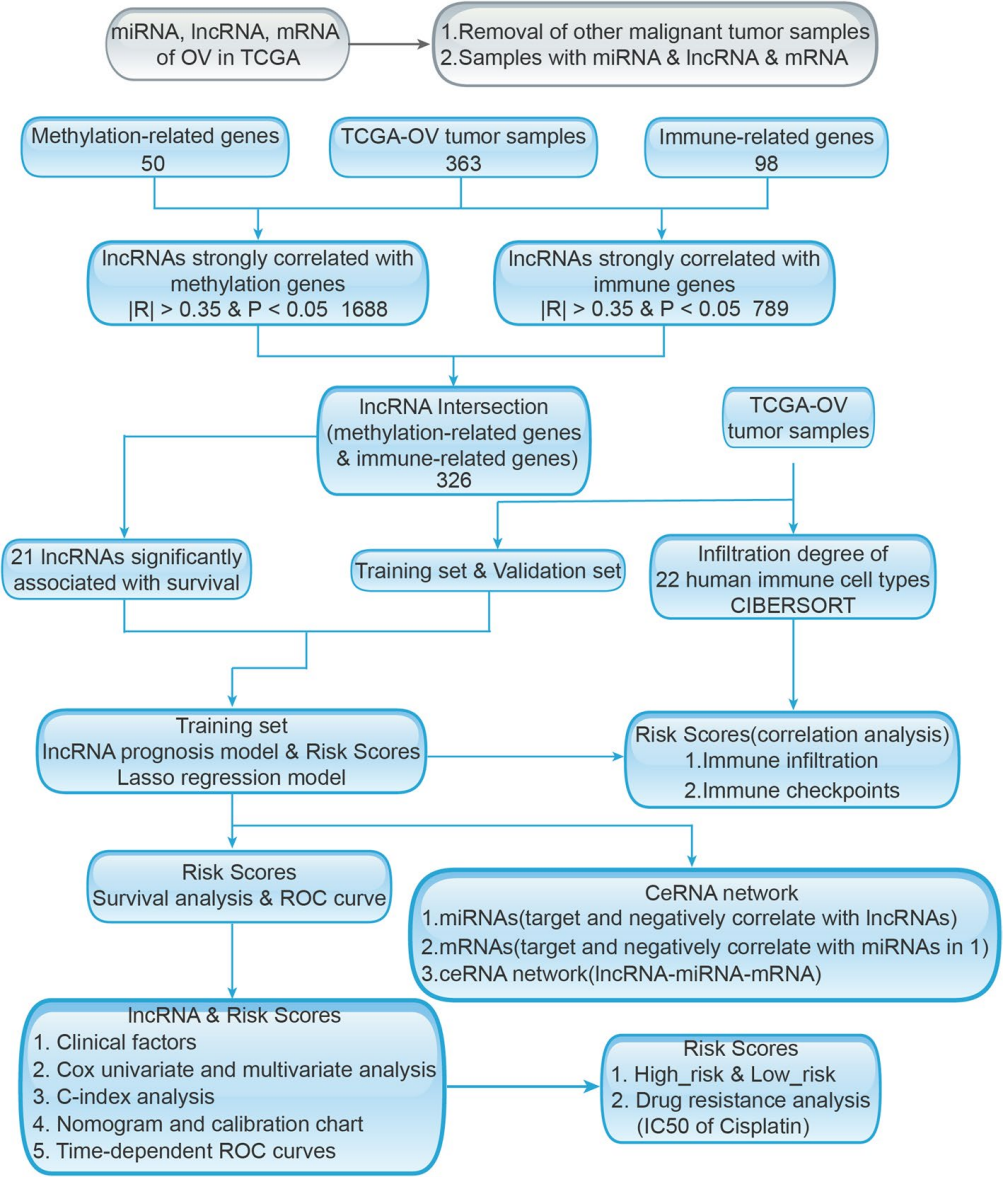
Workflflow chart of data generation and analysis

The flowchart of the study is depicted in Fig. 1. Step 1: To study the role of methylation modification and immune infiltration in predicting the prognosis of OC patients, we obtained OC clinical and transcriptome profile data from TCGA comprising 363 tumor samples. Patients were randomly divided into a training set (182 patients) and a validation set (181 patients). The eligible patients sampled in the analysis were required to meet the following criteria simultaneously: 1) the primary disease was diagnosed as ovarian cancer, removing patients who have ever been affected by other malignant tumors; 2) had available miRNA, mRNA and lncRNA sequencing data; 3) Patients with complete follow-up information including survival time, survival status, outcome, age, sex, clinical stage, and grade were selected to match with their RNA seq data. The main outcome of our study was overall survival. Patients without survival information were removed for further evaluation. Then, a list of 50 recognized methylation regulators was extracted from previous studies. The list of 98 immune-related genes was obtained from the literature related to OC immunity [38]. Step 2: The data were consolidated and used for coexpression analysis between the lncRNAs and the methylation regulators or the immune-related genes. The coincident lncRNA genes were selected from the two datasets according to relevant conditions. Step 3: Further screening for lncRNAs from coincident genes that were closely related to the OS of OC patients was performed. Step 4: A risk model was constructed and validated according to mrlncRNAs and irlncRNAs in OC patients. Step 5: The relationship between the prognostic models and immune infiltration, checkpoint expression, drug resistance and IC50 of cisplatin was analyzed. Step 6: The ceRNA network centered on the lncRNAs within the signature was constructed.

### Identification of mrlncRNAs and irlncRNAs in patients with OC and determination of the intersection of the two sets of lncRNAs

**Fig. 2:**
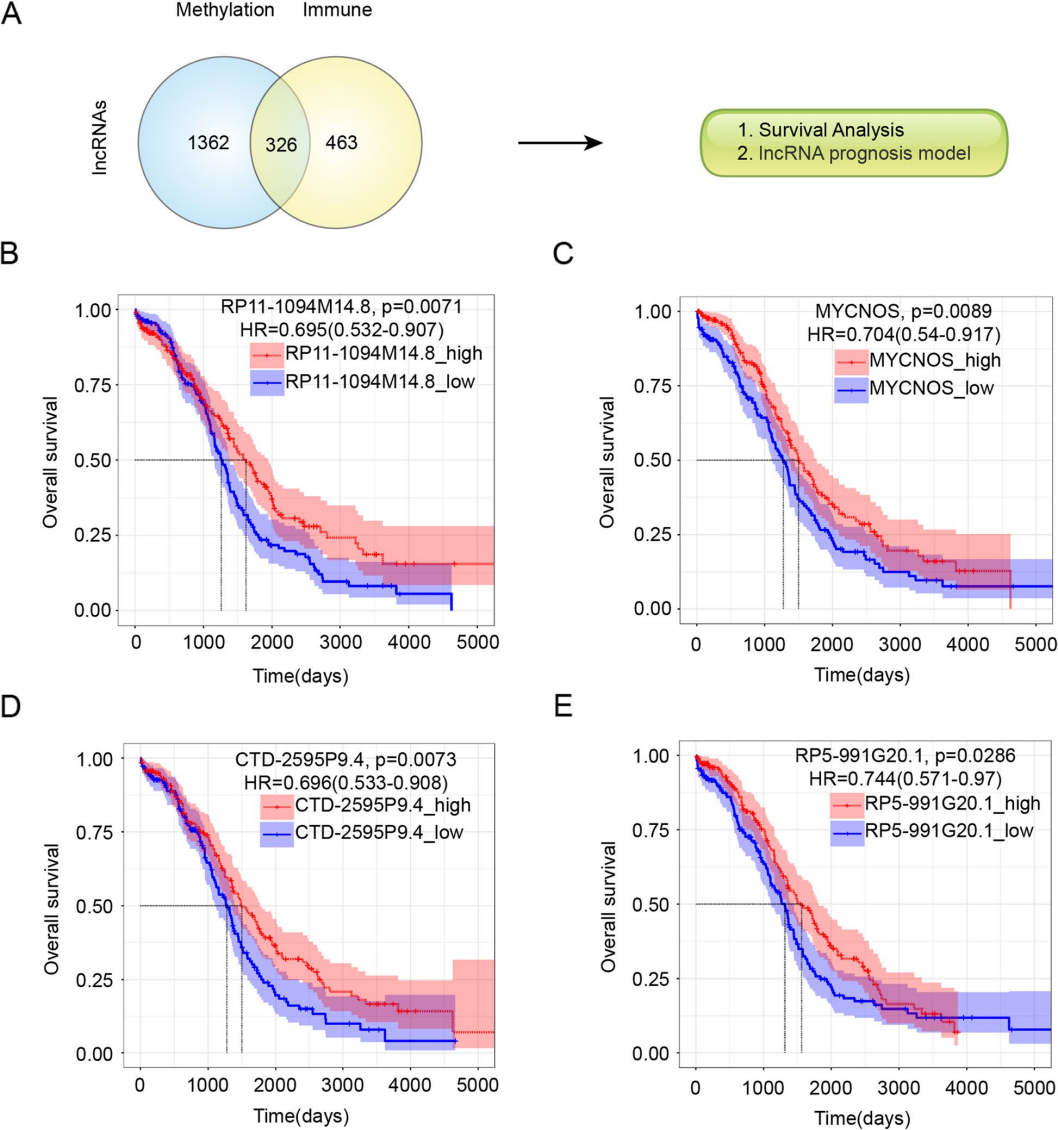
Survival analysis of lncRNAs in intersection. **A** Venn graph for intersection of lncRNAs associated with both methylation-related genes and immune-related genes. Kaplan–Meier survival curves of overall survival times between the high-expression and low-expression group of RP11-1094M14.8 (**B**), MYCNOS (**C**), CTD-2595P9.4 (**D**), RP5-991G20.1 (**E**)

We constructed a prognostic model with lncRNAs because lncRNAs display higher specificity to biological states than coding RNAs. Considering the prominent function of methylation regulators in regulating posttranscriptional modification, we evaluated the interplay between the methylation regulators and lncRNA transcriptome profiles of OC by weighted gene coexpression network analysis to identify mrlncRNAs, and a network of relationships between methylation regulators and the main mrlncRNAs was depicted. This analysis identified 1688 mrlncRNAs according to a correlation coefficient |Pearson R|> 0.35 and *p* < 0.05 (Supplementary Table 4). The same analysis was used for the lncRNAs and immune-related genes, which identified 789 irlncRNAs (Supplementary Table 5). The Venn diagram shows the intersection of the mrlncRNA gene set and irlncRNA gene set (Fig. 2A). Surprisingly, we obtained a total of 326 coincident lncRNA genes (Supplementary Table 6). This provided supporting evidence for the strong correlation between immunity and methylation. Next, we attempted to depict the prognostic value of the 326 overlapping lncRNAs by carrying out univariate Cox regression analysis. According to the associations of lncRNAs with the OS of patients, 21 of these lncRNAs were significantly correlated with OS (p < 0.05), including RPS12P23, AC004540.5, EXOC3-AS1, RP11-488L18.6, AC004540.4, ZNF32-AS1, MEIS1-AS3, DUX4L50, RP4-665N4.4, MYCNOS, AP000662.4, RP11-15E18.1, RP5-991G20.1, LCMT1-AS1, RP1-228H13.5, AC145343.2, CTD-2132N18.2, RP11-1094M14.8, CTD-2561J22.5, BNIP3P17, and CTD-2595P9.4 (Figs. 2B-E and S 1A-Q). Here, we identified the top eight lncRNAs, as shown in the Kaplan‒Meier (KM) survival curves (Figs. 2B-E and S 1A-D). High expression of EXOC3-AS1, RP11-1094M14.8, CTD-2595P9.4, MYCNOS, AP000662.4, AC145343.2, and ZNF32-AS1 indicated longer survival time, while high expression of CTD-2132N18.2 indicated shorter OS, which further confirmed the prognostic value of these 8 mrlncRNAs/irlncRNAs in patients with OC. Furthermore, we revealed the relationship between methylation regulators and the 8 lncRNAs through a heatmap. Interestingly, all 8 lncRNAs were positively correlated with methylation regulators. As shown in Figure S 2A, the 10 methylation regulators, including writers (METTL4, TRDMT1, TET1, TET2, PCIF1, METTL16, TET3, RBMX), readers (ALYREF), and erasers (TET2, FTO, TET3, TET1), demonstrated a close connection with 8 mrlncRNAs/irlncRNAs. Similarly, these 8 mrlncRNAs/irlncRNAs were significantly correlated with immune-related genes, as shown in Figure S 2B.

### Construction and validation of a risk model according to mrlncRNAs and irlncRNAs in OC patients

**Fig. 3:**
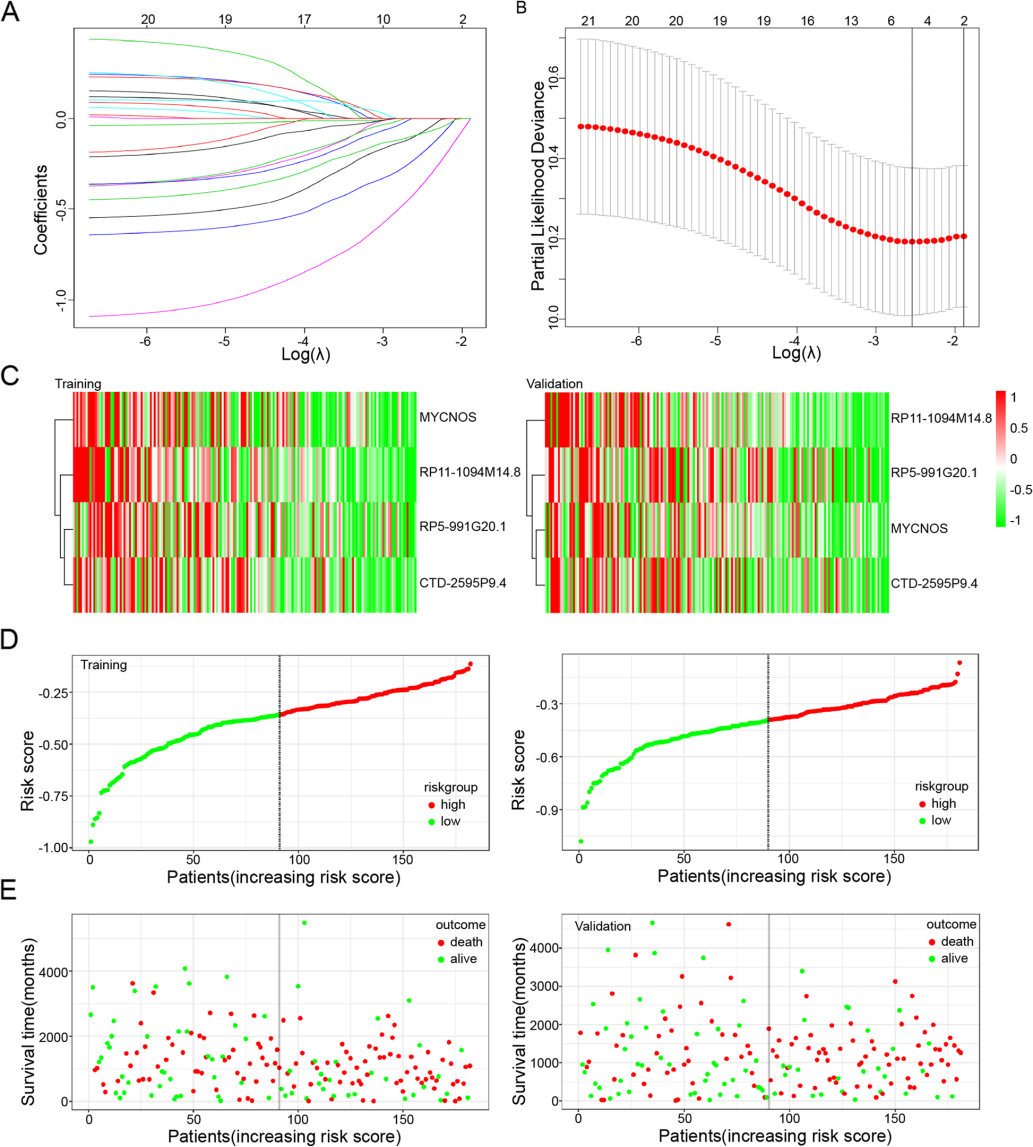
Establishment and validation of a 4 lncRNAs model. **A** LASSO Cox analysis of 21 lncRNA in intersection. **B** λ selection diagram. The two dotted lines indicated two particular values of λ. The left side was λmin and the right side was λ1se. The λmin was selected to build the model for accuracy in our study. **C** Clustering analysis heatmap shows the display levels of the 4 lncRNAs for each patient in the testing set. **D** Distribution of 4 lncRNAs model-based risk score for training set (left) and validation set (right). **E** Patients’ survival status in training set (left) and validation set (right). The x-axis is the patient ranking in ascending order by the ferroptosis risk score; the y-axis is the survival time. The red dots represent patients who died, and the green dots represent the surviving patients

Subsequently, we randomly divided the entire TCGA set into a training set (*n* = 182) and a validation set (*n* = 181). The training set was utilized to construct mrlncRNA and irlncRNA risk models. Then, LASSO Cox regression was performed in the training set, and 4 mrlncRNAs and irlncRNAs (MYCNOS, CTD-2595P9.4, RP11-1094M14.8, and RP5-991G20.1) highly correlated with OS were incorporated into a Cox proportional hazard model to construct a prognostic risk model when log (lambda) was between -2 and -3 (Fig. 3A). The regression coefficients are shown in Fig. 3B. In addition, the expression of MYCNOS, CTD-2595P9.4, RP11-1094M14.8, and RP5-991G20.1 in OC tumor tissue was demonstrated in both the training and validation sets (Fig. 3C). We sought to determine the potential prognostic capability of the 4 mrlncRNA and irlncRNA risk models in predicting OC patient OS. The training and validation sets were applied to validate the risk model. We first calculated the risk score of each patient according to the coefficients and expression of 4 mrlncRNAs and irlncRNAs and categorized OC patients into two subgroups (low-risk score and high-risk score groups) based on the median risk score (Fig. 3D). The distributions of the risk score and patient survival status are presented by scatter plots (Fig. 3E). From the results, it was obvious that the model presented good prediction power, and low-risk patients had better survival status than high-risk patients in both the training and validation sets.

### Verification of the grouping ability of the mrlncRNA and irlncRNA models

**Fig. 4:**
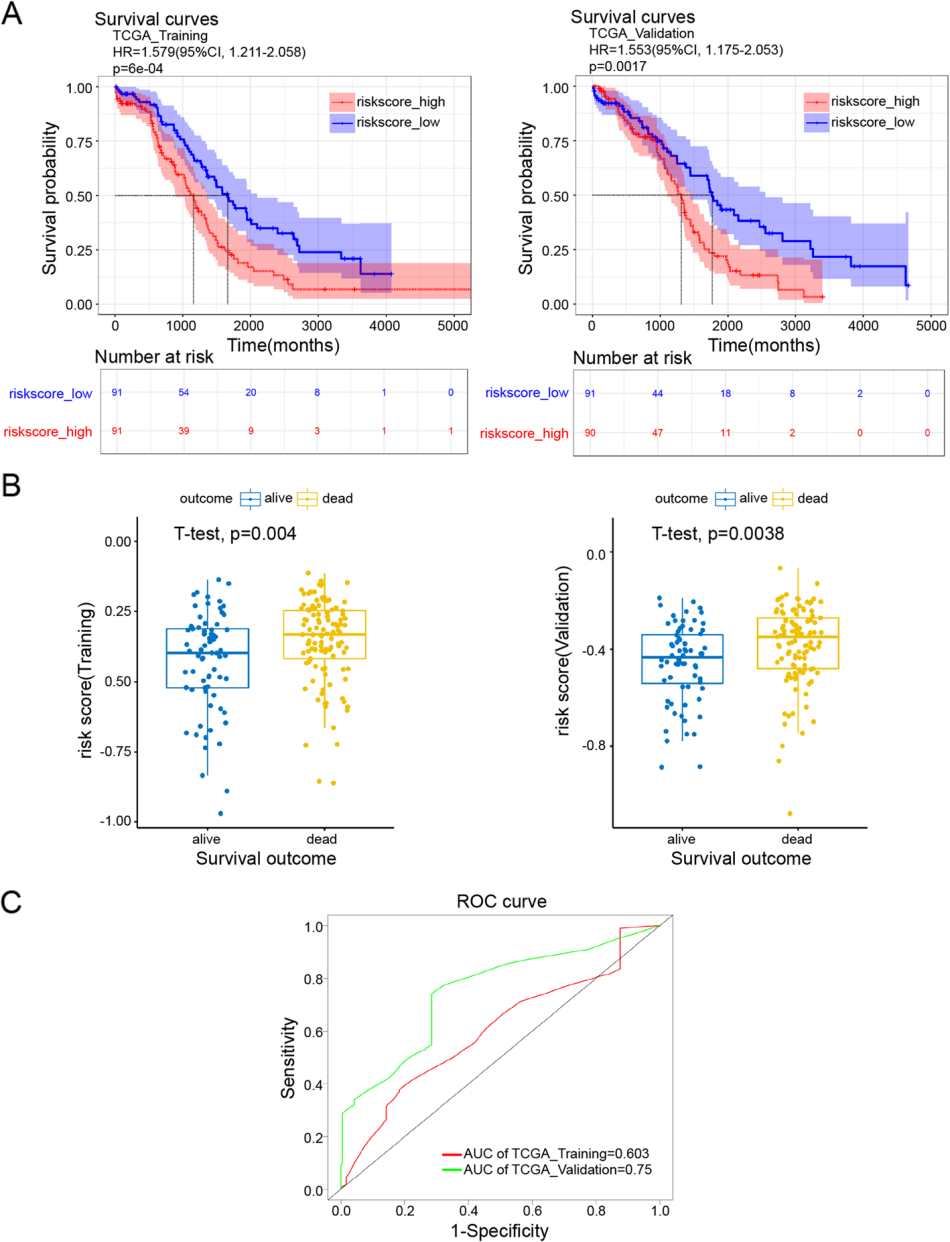
Survival analysis and ROC curve of training set and validation set. **A** Kaplan–Meier survival of training set (left) and validation set (right). **B** Risk histogram of training set (left) and validation set (right). **C** ROC curves measuring the predictive value of the risk score in the training set and validation set

Likewise, when we separated OC patients in the training and validation sets into high-risk and low-risk groups, the patients in the high-risk group had shorter survival times than those in the low-risk score group, as shown in the KM survival curve and distribution of risk scores (Fig. 4A). Scattergrams of survival status between subgroups were depicted, demonstrating that the low-risk score subgroup possessed a greater number of surviving patients than the high-risk score subgroup (Fig. 4B). The areas under the ROC curve (AUCs) for identification of the positive sample threshold of the training and validation sets were 0.603 and 0.75, respectively, which demonstrated that the mrlncRNA and irlncRNA risk models were able to precisely predict the survival of OC patients (Fig. 4C). In conclusion, the above results indicated the favorable predictive efficacy and sensitivity of the risk model.

### Comparison with clinical characteristics and construction of the prognostic nomogram

**Fig. 5:**
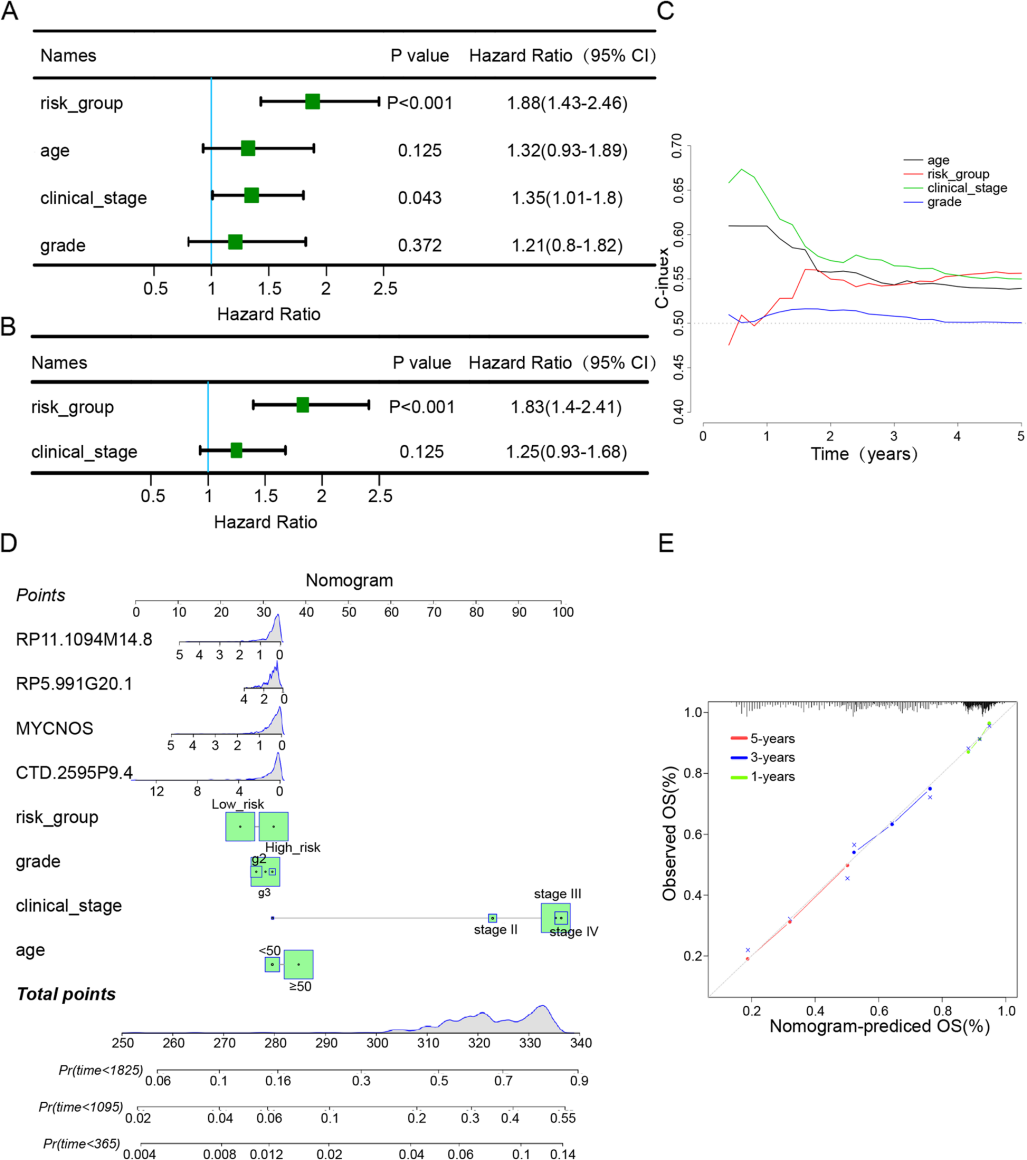
Independent prognostic factor evaluation and correlation with clinical characteristics. **A** Results of univariate analysis of clinical characteristics and RiskScore. **B** Results of multivariate analysis of clinical characteristics and RiskScore. **C** The prognostic performance was compared between the risk_group and different conventional clinical characteristics by calculating the C-index. **D** A nomogram constructed by RiskScore and clinical features. **E** A correction chart for survival rate of the nomogram

Furthermore, we used univariate and multivariable Cox regression analyses to determine whether mrlncRNA and irlncRNA model risk scores could serve as independent prognostic variables. We confirmed through univariate Cox regression analysis that risk score (HR = 1.88, 95% CI = 1.43–2.46, *P* < 0.001), age (HR = 1.32, 95% CI = 0.93–1.89, *P* = 0.125), clinical stage (HR = 1.35, 95% CI = 1.01–1.8, *P* = 0.043), and grade (HR = 1.21, 95% CI = 0.8–1.82, P = 0.372) exhibited obvious significant differences (Fig. 5A). The multivariate Cox regression analysis revealed that risk score (HR = 1.83, 95% CI = 1.4–2.41, *P* < 0.001) and clinical stage (HR = 1.25, 95% CI = 0.93–1.68, *P* = 0.125) were independent prognostic factors for OC (Fig. 5B). Additionally, to evaluate the stability and accuracy of the risk prognostic model, we calculated and compared the concordance index between classic clinical characteristics for predicting patient prognosis and the prognostic model. With increasing time, the concordance index of the risk score was always greater than that of other clinical factors (Fig. 5C), suggesting that the risk score could better forecast the prognosis of OC. To more intuitively predict the prognosis of OC patients in the clinic, a prognostic nomogram comprising the risk group, expression level of four IRLs (MYCNOS, CTD-2595P9.4, RP11-1094M14.8, and RP5-991G20.1) and clinical risk characteristics was fabricated to predict the 1-, 3-, and 5-year OS incidences for individual patients (Fig. 5D). In comparison with clinical factors such as grade and age, the risk grade of the prognostic model showed better predictive ability in the nomogram. The calibration curves showed that the predicted rates when compared to the observed 1-, 3-, and 5-year OS rates featured ideal consistency (Fig. 5E).

### Correlation of the infiltration of different immune cell populations with the prognostic model

**Fig. 6:**
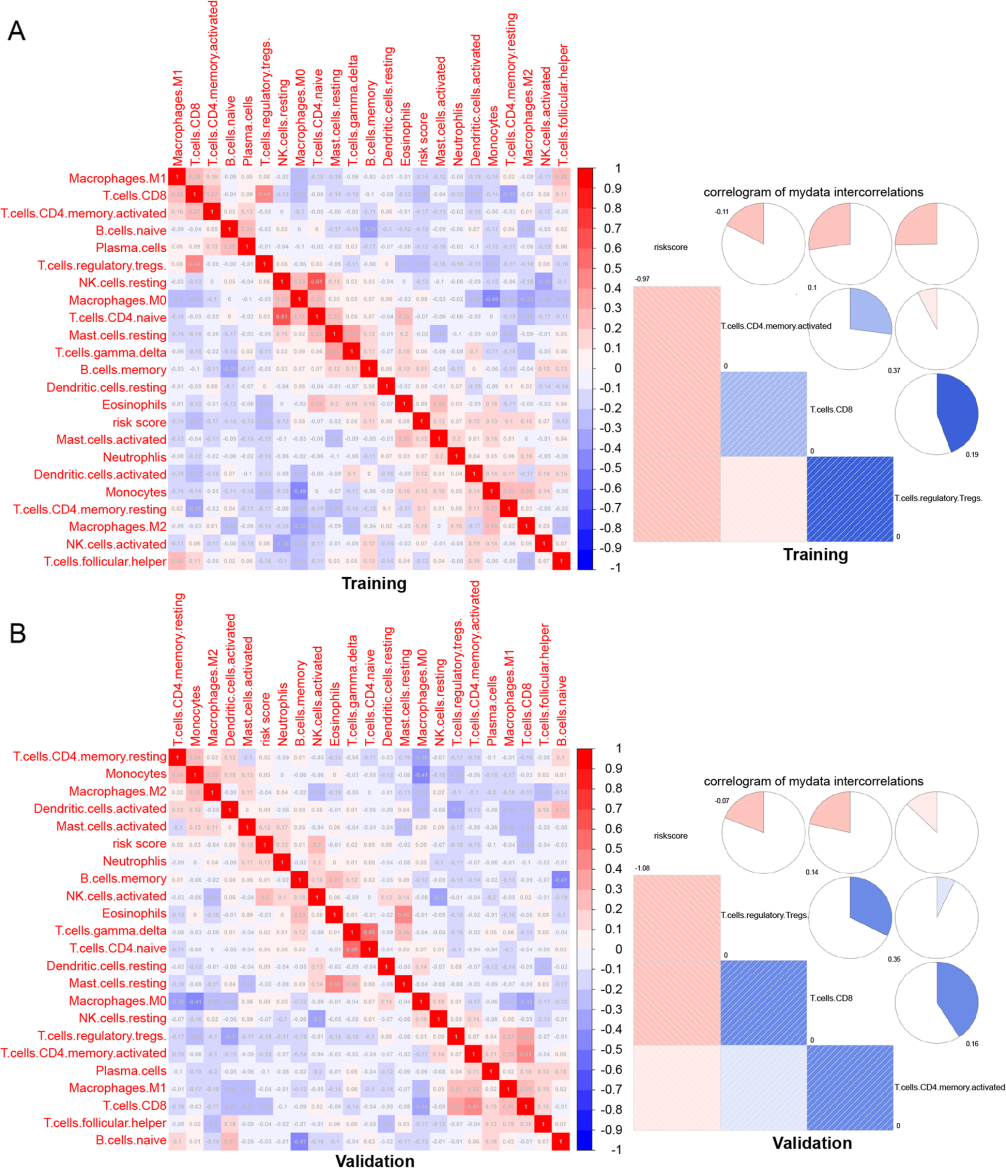
Correlation analysis between riskcore and immune cell infiltration. **A**, **B** Correlation matrix of all 22 immune cells (left) and 3 immune cells significantly related to riskcore (right) in training set (**A**) and validation set (**B**)

Multiple studies have reported an association between the extent of infiltrating immune cells and the prognosis of OC [41, 42]. To analyze the connection between the immune prognostic risk model and immune cell infiltration, the CIBERSORT algorithm was employed to investigate the expression of 22 subpopulations of immune cells in OC tissues. The correlation coefficient heatmap visualized the interaction of immune cell infiltration in the TIME, and the correlation of 22 subpopulations of immune cells with the risk score in each tissue sample was denoted. Obviously, the infiltration extent of many immune cell populations was associated with risk score in the training group and validation group, as shown in Fig. 6A and B. In the training group, we found that monocytes and M2 macrophages showed a strong positive correlation with the risk score. The infiltration of CD8 + T cells, Treg cells, M1 macrophages, naive B cells and CD4 + cells was significantly negatively related to the risk score in OC tissue (Fig. 6A). Consistently, in the training group, patients with greater infiltration of CD8 + T cells, Treg cells and CD4 + cells had a lower risk score and a better prognosis (Fig. 6B). Overall, the results suggest that infiltration of CD8 + T cells, Treg cells and CD4 + cells play an important role in affecting prognosis and survival of OC. Moreover, these results also suggest that the risk model could indirectly the correlation immune response of OC. This means that it may be possible to significantly stratify the immune response of patients with the prognostic model.

### Differential expression of immune checkpoints between the high-risk and low-risk groups

**Fig. 7:**
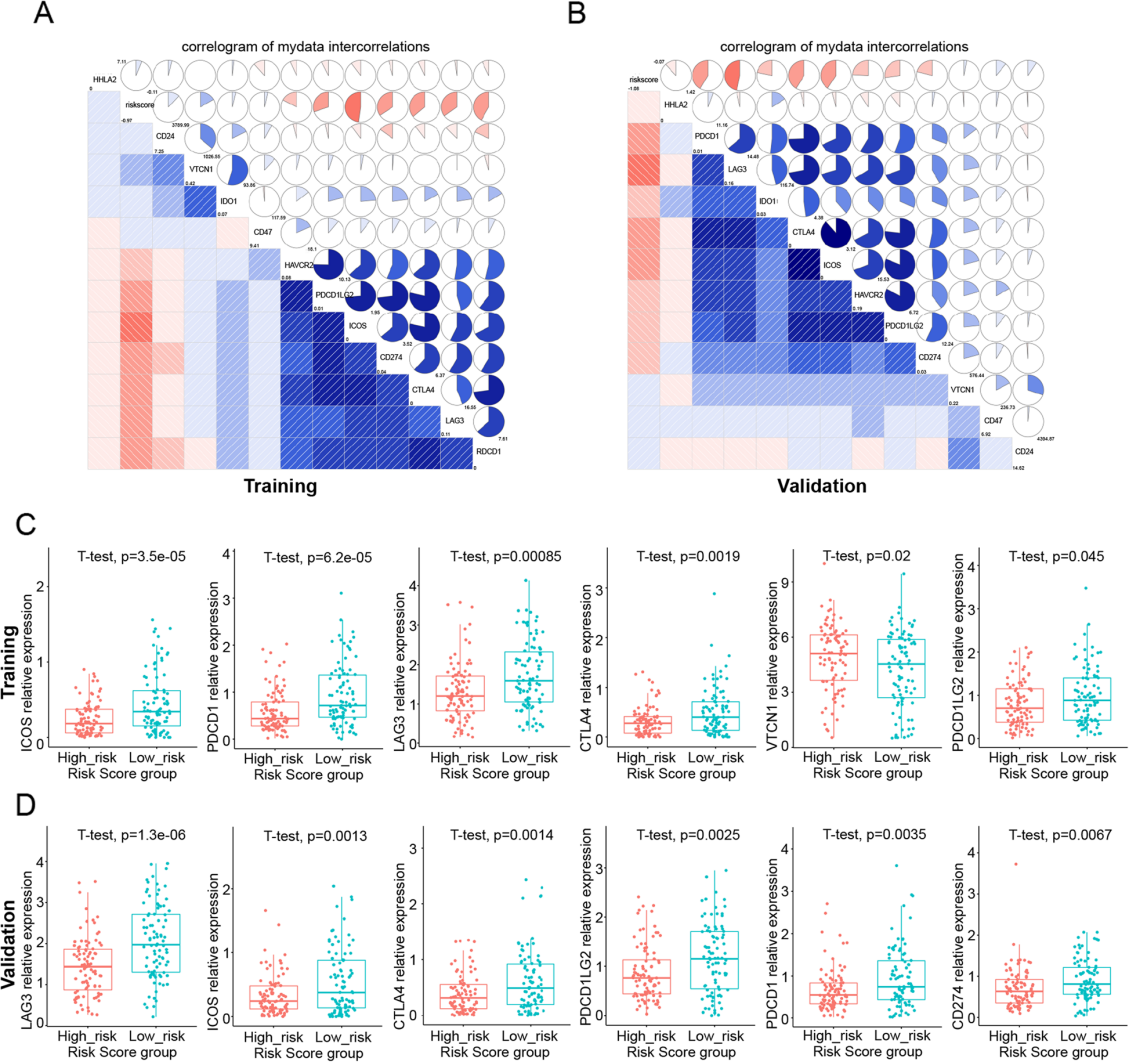
Correlation analysis between riskcore and immune checkpoint. **A**, **B** Correlation matrix of immune checkpoints associated with ovarian cancer in training set (**A**) and validation set (**B**). **C**, **D** Correlation histogram of 6 immune checkpoints significantly related to riskscore in training set (**C**) and validation set (**D**)

Considering the importance of immune checkpoints in tumor immunotherapy and prognosis, we subsequently investigated the relationship between the expression of 12 immune checkpoints and the risk score of the predictive models, as shown in Fig. 7A and B. The risk score showed a negative correlation with PDCD1, CD274, PDCD1LG2, CTLA4, ICOS, HAVCR2, and LAG3. However, a positive correlation was observed in VTCN1 expression in the training group (Figs. 7C and S 3A). The results showed that the low-risk group manifested higher expression of PDCD1, CD274, PDCD1LG2, CTLA4, ICOS, HAVCR2, and LAG3 and lower expression of VTCN1 than the high-risk group. Similarly, in the validation set, the expression of PDCD1, CD274, PDCD1LG2, CTLA4, ICOS, HAVCR2, and LAG3 was inversely correlated with the risk score (Figs. 7D and S 3B). The above results illustrate the predictive power of the prognostic model in predicting the expression level of immune checkpoints in OC patients, which is closely related to immunotherapy responses.

### Construction of a ceRNA network and prediction of sensitivity to chemotherapy drugs based on the mrlncRNA and irlncRNA signatures

**Fig. 8:**
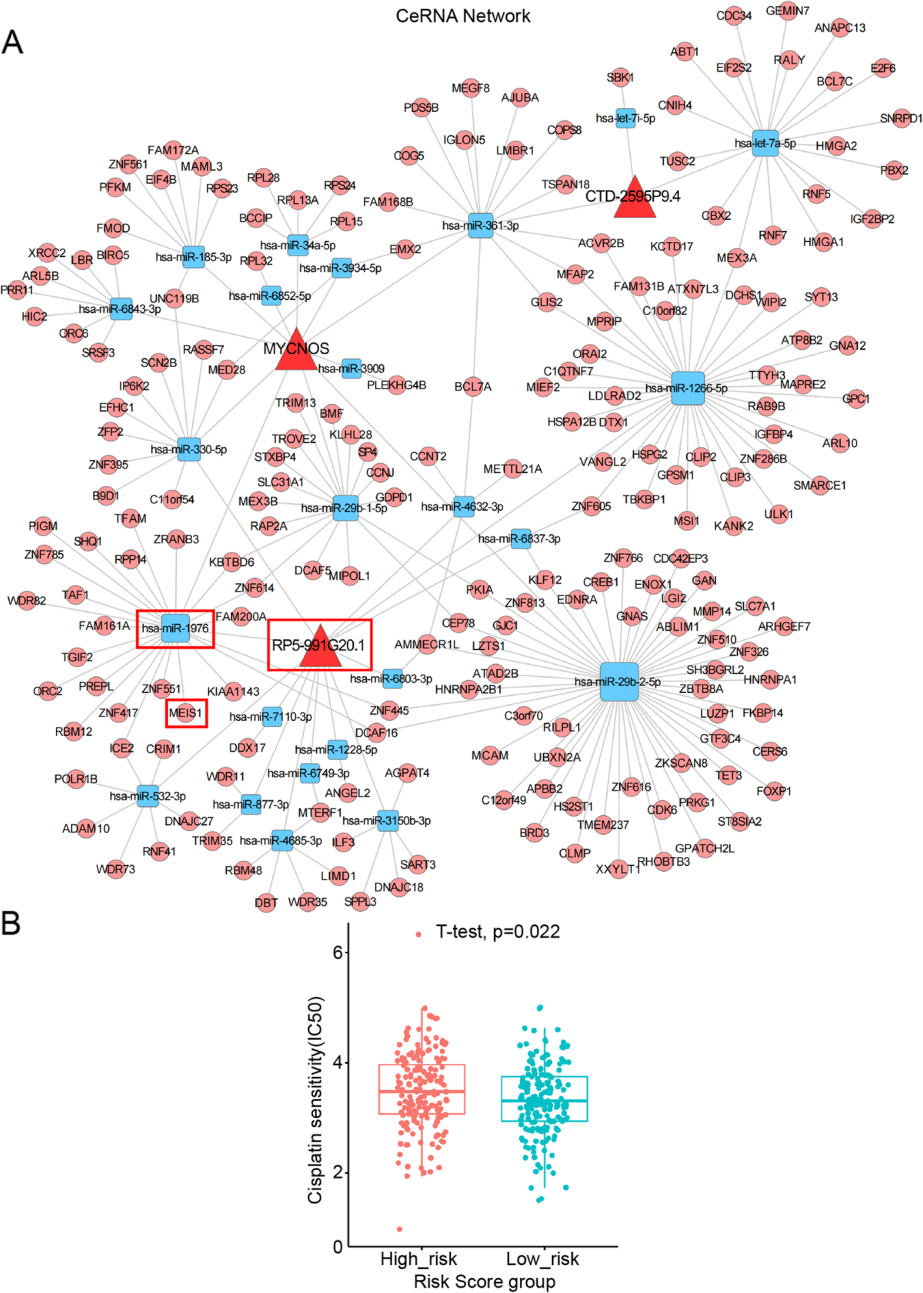
ceRNA network and Prediction of the Sensitivity to Cisplatin. **A** Red triangle denotes deregulated lncRNA. Blue square denotes miRNA. Pink circle denotes targeted mRNA. **B** The IC50 of cisplatin in high_risk group and low_risk group

Considering that the lncRNAs MYCNOS and RP11-1094M14.8 acted as sponges in the occurrence and development of tumors in multiple studies [43, 44], we constructed a mrlncRNA and irlncRNA ceRNA network to generate a reliable signaling axis to reveal the underlying causes of chemoresistance, poor prognosis and immune silencing in patients with OC. Next, ceRNA networks centered on mrlncRNAs and irlncRNAs were constructed by employing the miRanda algorithm and the PITA algorithm. First, miRNAs negatively correlated with MYCNOS, CTD-2595P9.4, RP11-1094M14.8, and RP5-991G20.1 were extracted from the OC sequencing profile by correlation analysis (Supplementary Table 7). Then, the potential target miRNAs of model lncRNAs were obtained by target gene prediction. Subsequently, the target mRNAs of miRNAs were generated in the same way (Supplementary Table 8). Finally, we constructed a ceRNA network composed of 3 lncRNAs, 24 miRNAs, and 207 mRNAs (Fig. 8A). To understand the biological functions of RNAs in the ceRNA network, we searched for relevant studies, and we were surprised to find that most of the mRNAs and miRNAs are involved in tumorigenesis, drug resistance and immune regulation, some play a critical role in OC progression and drug resistance. Ultimately, by synthesizing information from previous studies and the GEPIA database, we successfully determined a core regulatory axis, RP5-991G20.1/hsa-miR-1976/MEIS1, for subsequent research. In addition, these results suggested the potential role of prognostic risk models in predicting drug resistance. Therefore, we predicted the responses to cisplatin in each OC patient based on the gene expression profiles from the TCGA database. Our data showed that the IC50 levels of cisplatin in the low-risk group were significantly lower than those in high-risk group (Fig. 8B), indicating that OC patients in the low-risk group were more sensitive to cisplatin. The results demonstrated the predictive ability of mrlncRNA and irlncRNA signatures for the chemotherapy response of patients with OC.

### FTO regulates the chemoresistance of ovarian cancer cells through the RP5-991G20.1/hsa-miR-1976/MEIS1 signaling pathway

**Fig. 9:**
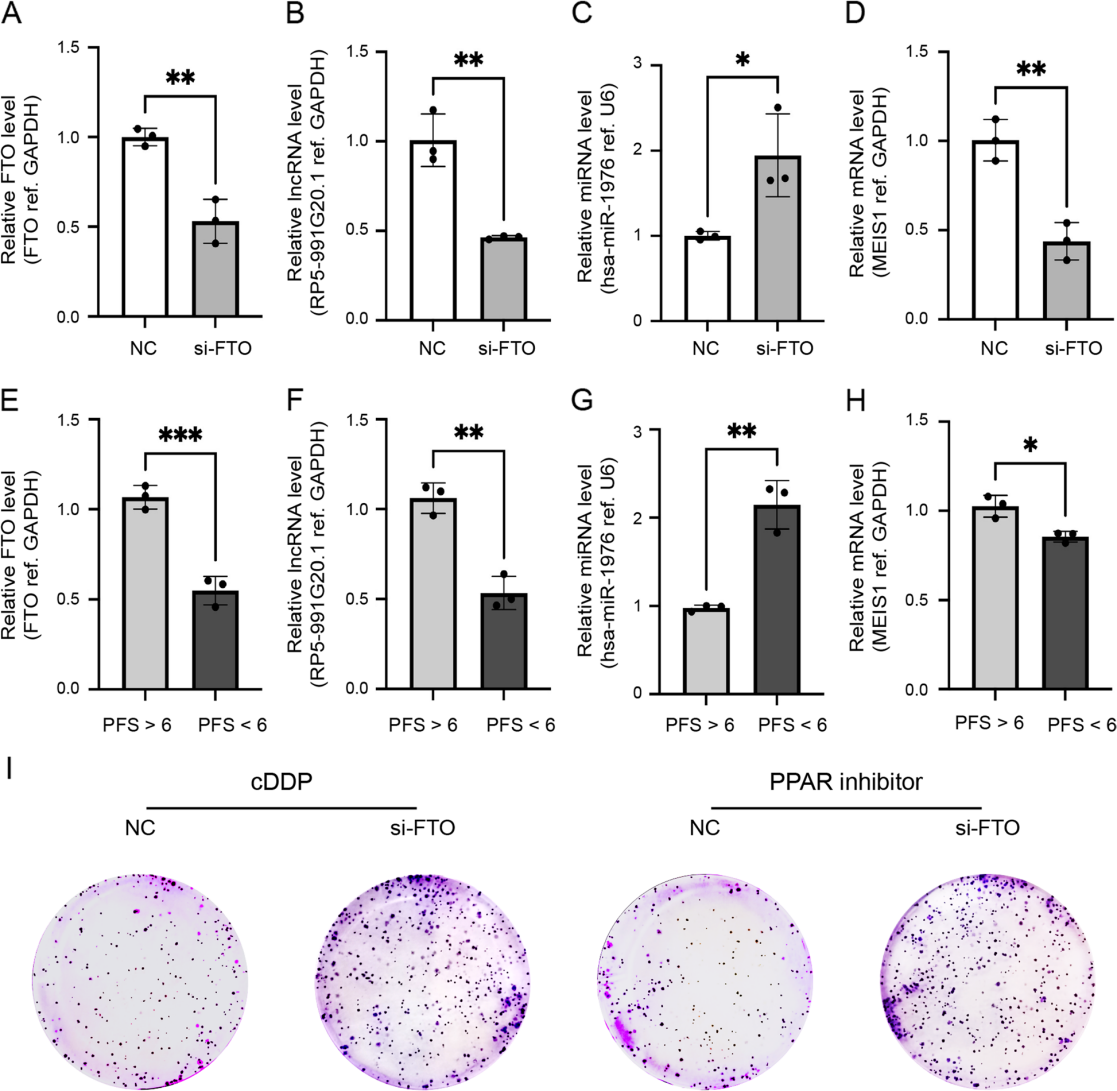
FTO sensitizes ovarian cancer cells to cDDP and PPAR inhibitor via RP5-991G20.1/hsa-miR-1976/MEIS1 axis. Relative level of FTO (**A**), relative lncRNA level of RP5-991G20.1 (**B**), relative miRNA level of hsa-miR-1976 (**C**) and relative mRNA level of MEIS1 (**D**) were detected in A2780 cells transfected with si-FTO. Relative level of FTO (**E**), relative lncRNA level of RP5-991G20.1 (**F**), relative miRNA level of hsa-miR-1976 (**G**) and relative mRNA level of MEIS1 (**H**) were detected in tumour specimens from ovarian cancer patients with PFS > 6 months vs. PFS < 6 months. Cell colonies assay (**I**) of A2780 cells transfected with si-FTO were shown

**Fig. 10:**
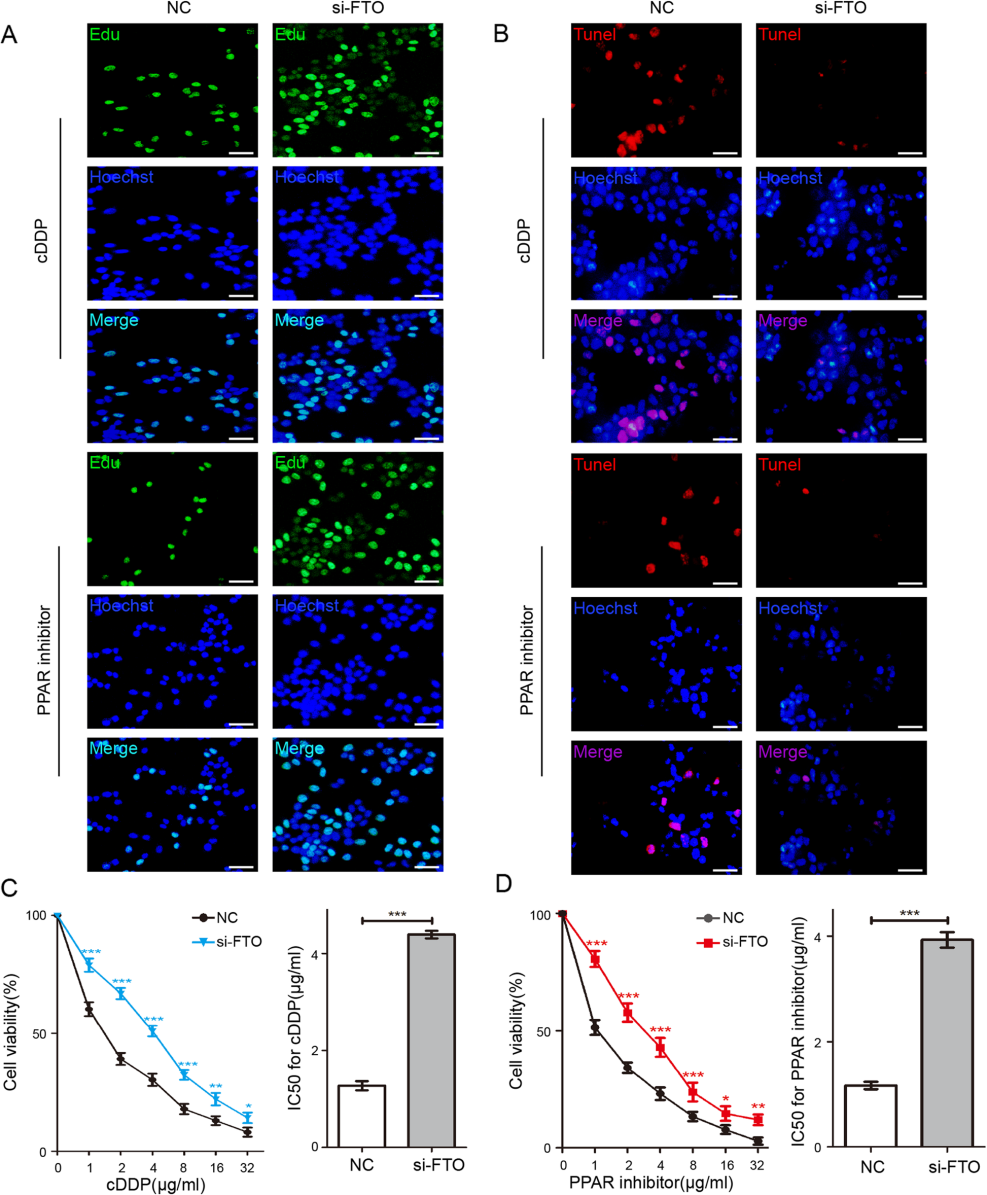
FTO increases the drug sensitivity of ovarian cancer cells to cDDP and PPAR inhibitors. Cell Edu assay (Scale bar: 200 μm) (**A**) and Tunel staining experiment (Scale bar: 200 μm) (**B**) of A2780 cells transfected with si-FTO were shown. Cell Viability (left panels) and IC50 (right panels) for cDDP (**C**) and PPAR inhibitor (**D**) in A2780 cells transfected with si-FTO. *p < 0.05, **p < 0.01, ***p < 0.001

To verify the function of mrlncRNAs and irlncRNAs in OC and to explore the regulatory function of the methylation regulator gene FTO on irlncRNAs RP5-991G20.1, hsa-miR-1976 and MEIS1, we constructed a FTO knockout A2780 cell model (Fig. 9A) and verified it by qRT-PCR in vitro. As shown in Fig. 9B-D, we detected the expression levels of RP5-991G20.1 and MEIS1 in each group, and compared with those in the control group, the expression levels of RP5-991G20.1 and MEIS1 in the FTO knockout group were significantly decreased. In contrast, the expression level of hsa-miR-1976 in the FTO knockout group was significantly higher than that in the control group and was negatively correlated with FTO. This result suggests that changes in the expression of FTO could affect the levels of RP5-991G20.1/hsa-miR-1976/MEIS1, supporting our hypothesis and subsequent studies. In addition, the expression levels of FTO, RP5-991G20.1 and MEIS1 were clearly increased in tumors obtained from patients with progression-free survival (PFS) > 6 months (clinically described as cDDP sensitive), and reduced in tumors obtained from patients with PFS < 6 months (cDDP resistant; Fig. 9E, F and H). In contrast, cDDP-resistant patients (PFS < 6) showed higher hsa-miR-1976 expression, and cDDP-sensitive patients (PFS > 6) exhibited lower hsa-miR-1976 expression (Fig. 9G). Considering the role of the demethylase FTO in regulating PPAR resistance in OC and the role of MEIS1 in suppressing oxaliplatin resistance and tumorigenesis in colorectal cancer, we performed colony forming, EdU and CCK-8 assays on A2780 cells with FTO knockdown to verify whether FTO could reverse OC cell proliferation and chemoresistance by modulating the RP5-991G20.1/hsa-miR-1976/MEIS1 signaling pathways. As shown in Figs. 9I and 10A-D, compared with the control group, FTO knockdown significantly enhanced the proliferation and cDDP and PPARi resistance of A2780 cells, simultaneously reducing the degree of apoptosis, indicating that FTO restrains the proliferation and drug resistance of OC cells, which further confirms the critical role of FTO in reversing ovarian cancer resistance and growth.

## Discussion

The immune system is an important barrier to disease surveillance and clearance. Immune silencing is recognized as a vital hallmark of tumor development, which is associated with multiple characteristics of cancer, such as chemoresistance, transfer and invasion [45]. Early studies indicated that alteration of immune cell infiltration status, modification of PD1/PD-L1 expression patterns, and polarization of immune cells contributed to the development of OC [46]. However, we have not yet found effective signaling pathways to improve prognosis and immunotherapy response in OC, and our study was designed to solve this problem.

With the rapid development of bioinformatics analysis technology, we attempted to explore the potential value of the combination of immunity and methylation in OC and signaling pathways that regulate tumor immune responses based on the maximum transcriptomic sample data that could be acquired from TCGA and GEO. Growing evidence suggests that the expression of immune-related genes shows great prognostic value regarding tumor progression, and the methylation modification of noncoding RNA plays an important regulatory role in a series of biological functions [47]. Thus, methylation and immune infiltration will greatly affect the outcome of patients with malignancy [48, 49]. Similarly, it will inevitably affect the prognosis and treatment response of patients with OC.

As we known, the ICI score for predicting the prognosis of OC patients and providing the potential response to immunotherapy, painting a novel picture of regulation of immune response and immunotherapy, confirmed its association with clinical outcome [14]. Here, Our study constructed a methylation- and immune-related risk model and ceRNA network. Moreover, we also explored the prognostic predictive ability of the risk model and its association with immune cell infiltration and assessed the reactivity of OC patients to chemotherapy. In this study, 4 mrlncRNAs and irlncRNAs were incorporated into a risk model. Kaplan‒Meier curves, ROC curves, Cox regression analysis, and nomograms showed that the risk model possessed excellent prediction ability and was an independent predictor of OC prognosis.

Emerging evidence has demonstrated that lncRNAs play an essential role in regulating immune cell infiltration, and some studies have also reported that methylation modification of lncRNAs regulates immune cell function in the tumor immune microenvironment [50, 51]. In addition, we investigated the fundamental effects of the risk score on the regulation of immune cell infiltration. Consistent with previous reports [52], the distribution of immune cell infiltration was significantly different between the low-risk and high-risk groups. Our study showed several interesting findings regarding the prognostic relevance of several well-known immune cells (e.g., CD8 + T cells, CD4 + T cells, and Tregs). CD8 + T cells and CD4 + T cells have been reported to be associated with better outcomes, whereas there are two different scenarios for Treg cells in OC. Our study indicated that CD8 + T cells and CD4 + T cells were associated with better outcomes among patients with low-risk OC but were associated with worse outcomes among patients with high-risk OC. Furthermore, CD8 + T cells are known to signify a favorable clinical outcome in a variety of tumors, including OC [53]. Therefore, combined with previous studies, we found that CD8 + T cells can predict the prognosis of OC patients [41]. The study also showed that the risk score was negatively correlated with the proportions of resting immune cells but positively associated with immunosuppressive cells, indicating that patients with low-risk scores were immunologically resting, while those with high-risk scores represented an immunosuppressive tumor microenvironment.

It has been reported that the response to anti-checkpoint blockade is affected by intertumoral infiltration of immune cells [54]. With the development of immune checkpoint inhibitors, immune-checkpoint blockade (ICB) immunotherapy has generated promising therapeutic results in tumors [55]. Unfortunately, the majority of OC patients do not respond to ICB treatment. Thus, we investigated the correlation of the risk scores, which are positively associated with immunosuppressive cells, and the expression of immune checkpoints to predict OC patients’ responses to immunotherapy. It has been reported that increased levels of immune checkpoints, such as PD-1 and CTLA-4, indirectly indicate preexisting T-cell activation, and patients might be more sensitive to ICI treatment [56]. Consistent with this conclusion, we achieved the same results in the training and validation sets: high expression of PDCD1, LAG3, ICOS, CTLA4, and CD274 indicated lower risk scores and better outcomes of OC. Consistently, when patients had lower risk scores and high expression of immune checkpoint genes, the infiltration of protective immune cells such as CD8 + T cells and CD4 + T cells was obviously enhanced. This result suggests that we can improve the immune evasion of tumor cells caused by the overexpression of immune checkpoints by altering immune cell infiltration in the tumor microenvironment.

Furthermore, in our research, a methylation and immune-related ceRNA regulatory network including 208 mRNAs, 24 miRNAs, and 3 lncRNAs was constructed to investigate the potential molecular mechanism of tumor immunity. Combined with the results of previous studies and the data from the GEPIA online database, we surprisingly found that the lncRNA RP5-991G20.1 within the network was significantly downregulated in OC. The findings echoed previous results that the high expression of RP5-991G20.1 indicated a longer OS of patients with OC. Moreover, the m6A demethylase FTO, which is positively correlated with lncRNA RP5-991G20.1, was reported to inhibit OC progression and stem cell self-renewal through demethylase activity [57]. Moreover, most of the participants within ceRNA networks are involved in drug resistance and immunomodulation of tumors [58]. Among them, the target gene of lncRNA RP5-991G20.1, MEIS1, is reported to promote the migration and chemotaxis of CD8 + T cells and indicate a favorable prognosis for patients with OC [59]. Therefore, we believe that the signaling pathway regulating the expression of RP5-991G20.1 and MEIS1 through the alteration of FTO will have a significant impact on the prognosis of OC.

Then, we validated the FTO/RP5-991G20.1/hsa-miR-1976/MEIS1 pathway in A2780 cells, and the results showed that FTO positively regulates the expression of RP5-991G20.1 and MEIS1 and is negatively correlated with the expression of hsa-miR-1976. Moreover, low-grade epithelial ovarian cancer (EOC) and high-grade EOC are two very different types of cancer, which is not well described in the TCGA database. To compensate for this deficiency, we selected low-grade EOC as the sensitive group (PFS > 6) and high-grade EOC as the drug-resistant group (PFS < 6). And then, we found that the expression levels of FTO, RP5-991G20.1 and MEIS1 were clearly increased in the sensitive group. However the hsa-miR-1976 had an opposite trend. A previous study reported that MEIS1 promoted the migration and chemotaxis of CD8 + T cells in OC [59], while RP5-991G20.1 was significantly downregulated within the tumor tissue of OC. These results suggest that these signaling pathways can be a new tool to improve the chemoresistance and immune therapeutic response of OC.

Our study is the first to investigate the molecular mechanism affecting the prognosis of OC from the perspective of methylation and immunity. Additionally, mrlncRNA and irlncRNA prognostic models possessing high predictive ability for survival in OC were constructed, and a ceRNA network was developed based on mrlncRNA and irlncRNA that may provide novel ideas for the study of OC.

## Conclusion

In conclusion, we utilized bioinformatic approaches analyzing TCGA datasets to construct an immune risk prognostic model to predict the prognosis and therapy response of OC patients. Finally, we identified a core regulatory axis, FTO/RP5-991G20.1/hsa-miR-1976/MEIS1, that may play a pivotal role in regulating immune cell infiltration and prognosis in OC patients. In addition, we identified novel targets and regulatory pathways for antitumor immunotherapy.

### Supplementary Information

**Additional file 1:** **Supplementary Table 1.** The 363 TCGA-OV samples list.

**Additional file 2:** **Supplementary Table 2.** The list of 50 recognized methylation regulators.

**Additional file 3:** **Supplementary Table 3.** The 98 immune related-genes list.

**Additional file 4:** **Supplementary Table 4.** The 1688 mrlncRNAs list.

**Additional file 5:** **Supplementary Table 5.** The 789 irlncRNAs list.

**Additional file 6:** **Supplementary Table 6.** The 326 coincident lncRNA genes list.

**Additional file 7:** **Supplementary Table 7.** miRNAs negatively correlated with 4 lncRNAs.

**Additional file 8:** **Supplementary Table 8.** mRNAs negatively correlated with miRNAs.

**Additional file 9:** **Figure S1.** Survival analysis of lncRNAs in intersection. (A-Q) Kaplan-Meier survival curves of overall survival times between the high-expression and low-expression group of EXOC3-AS1 (A), CTD-2132N18.2 (B), AP000662.4 (C), AC145343.2 (D), ZNF32-AS1 (E), MEIS1-AS3 (F), AC004540.4 (G), RP1-228H13.5 (H), RP11-15E18.1 (I), AC004540.5 (J), RP11-488L18.6 (K), CTD-2561J22.5 (L), DUX4L50 (M), RPS12P23 (N), RP4-665N4.4 (O), LCMT1-AS1 (P), BNIP3P17 (Q).

**Additional file 10:** **Figure S2.** lncRNAs strongly correlated with methylation genes and immune genes. (A) The top 20 lncRNAs strongly correlated with methylation genes. (B) The top 20 lncRNAs strongly correlated with immune genes. The process used the criteria of |Pearson R| >0.35 and *p* <0.05.

**Additional file 11:** **Figure S3.** Correlation histogram of immune checkpoints. Correlation histogram of immune checkpoints related to riskscore in training set (A) and validation set (B).

## Data Availability

The raw data will be made available by the authors without undue reservation.

## Abbreviations

OC: Ovarian cancer
lncRNA: Long noncoding RNA
irlncRNAs: Immune-related lncRNAs
mrlncRNAs: Methylation-related lncRNAs
OS: Overall survival
ICI: Immune cell infiltration
TMB: Tumor mutation burden
m1A: N1-methyladenosine
m6A: N6-methyladenosine
m5C: 5-Methylcytosine
m7G: N7-methylguanosine
m6Am: N6,2'-O-dimethyladenosine
hm5C: 5-Hydroxymethylcytosine
TCGA: The Cancer Genome Atlas database
DEGs: Differentially expressed genes
DEIRGs: Differentially expressed immune-related genes
IC50: Half maximal inhibitory concentration
cDDP: Cisplatin
PPAR: Peroxisome proliferator-activated receptor
