# Correction: A methylation‑ and immune‑related lncRNA signature to predict ovarian cancer outcome and uncover mechanisms of chemoresistance

**DOI:** 10.1186/s13048-024-01438-9

**Published:** 2024-05-24

**Authors:** Lu Chen, Wujiang Gao, Li Lin, Chunli Sha, Taoqiong Li, Qi Chen, Hong Wei, Meiling Yang, Jie Xing, Mengxue Zhang, Shijie Zhao, Wenlin Xu, Yuefeng Li, Lulu Long, Xiaolan Zhu

**Affiliations:** 1 Reproductive Medicine Center, The Fourth Affiliated Hospital of Jiangsu University, No. 20, Zhengdong Road, Zhenjiang City, 212001 Jiangsu Province China; 2 Department of Gynaecology and Obstetrics, Taixing People’s Hospital, Taixing, Jiangsu China; 3 Department of Gynaecology and Obstetrics, Yangzhou First People’s Hospital, Yangzhou, Jiangsu China; 4 Department of Gynaecology and Obstetrics, The First People’s Hospital of Nantong City, Nantong, Jiangsu China; 5 Medical School, Jiangsu University, No. 301, Xuefu Road, Zhenjiang City, 212031 Jiangsu Province China; 6 Oncology Department, Affiliated People’s Hospital of Jiangsu University, No. 8, Dianli Road, Zhenjiang City, 212001 Jiangsu Province China

**Correction: J Ovarian Res 16, 186 (2023)**

10.1186/s13048-023-01260-9

Following publication of the original article [1], an error was spotted in Fig. 4a where the x-axis should say ‘time (months)’ rather than ‘time (mouths)’. The correct images were shown below.

Incorrect figure:
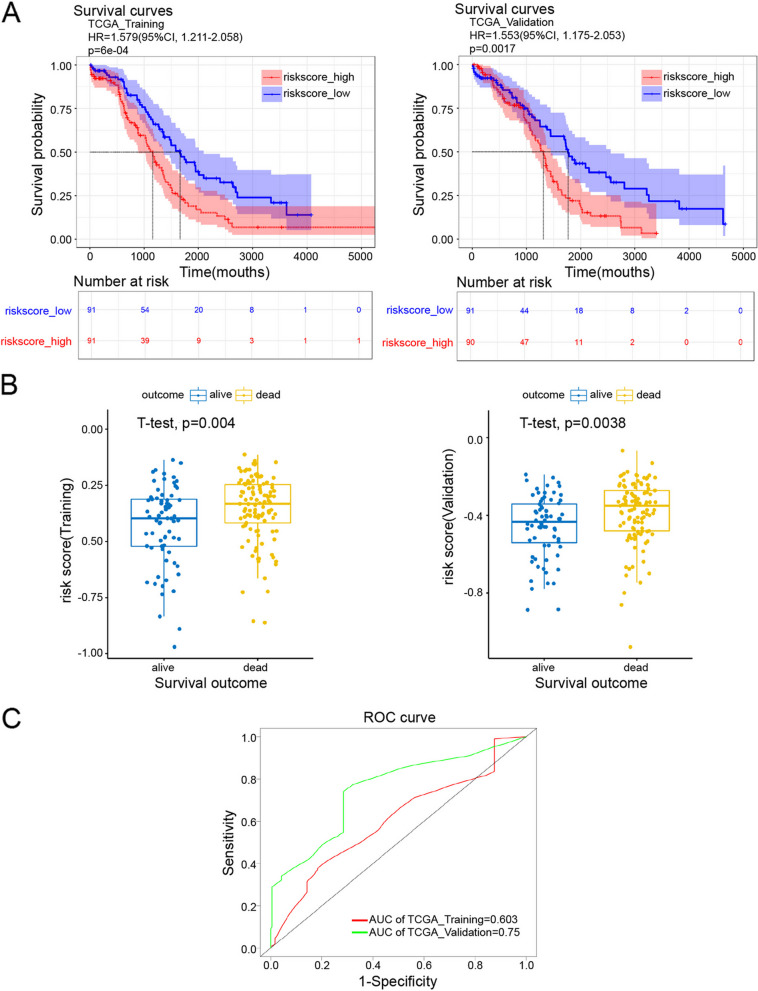


Correct figure:
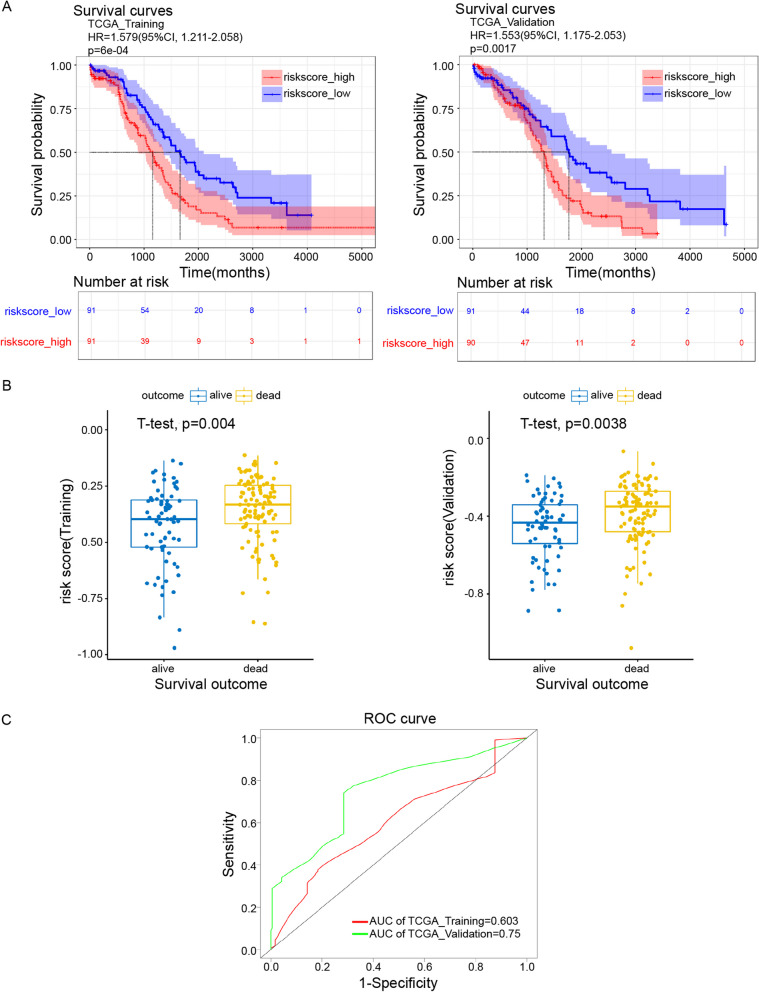


The original article has been corrected.
