# Supplementary figures and images for: A methylation- and immune-related lncRNA signature to predict ovarian cancer outcome and uncover mechanisms of chemoresistance
Source: J Ovarian Res. 2023 Sep 6;16:186. doi: 10.1186/s13048-023-01260-9 (PMC10483746; doi:10.1186/s13048-023-01260-9)

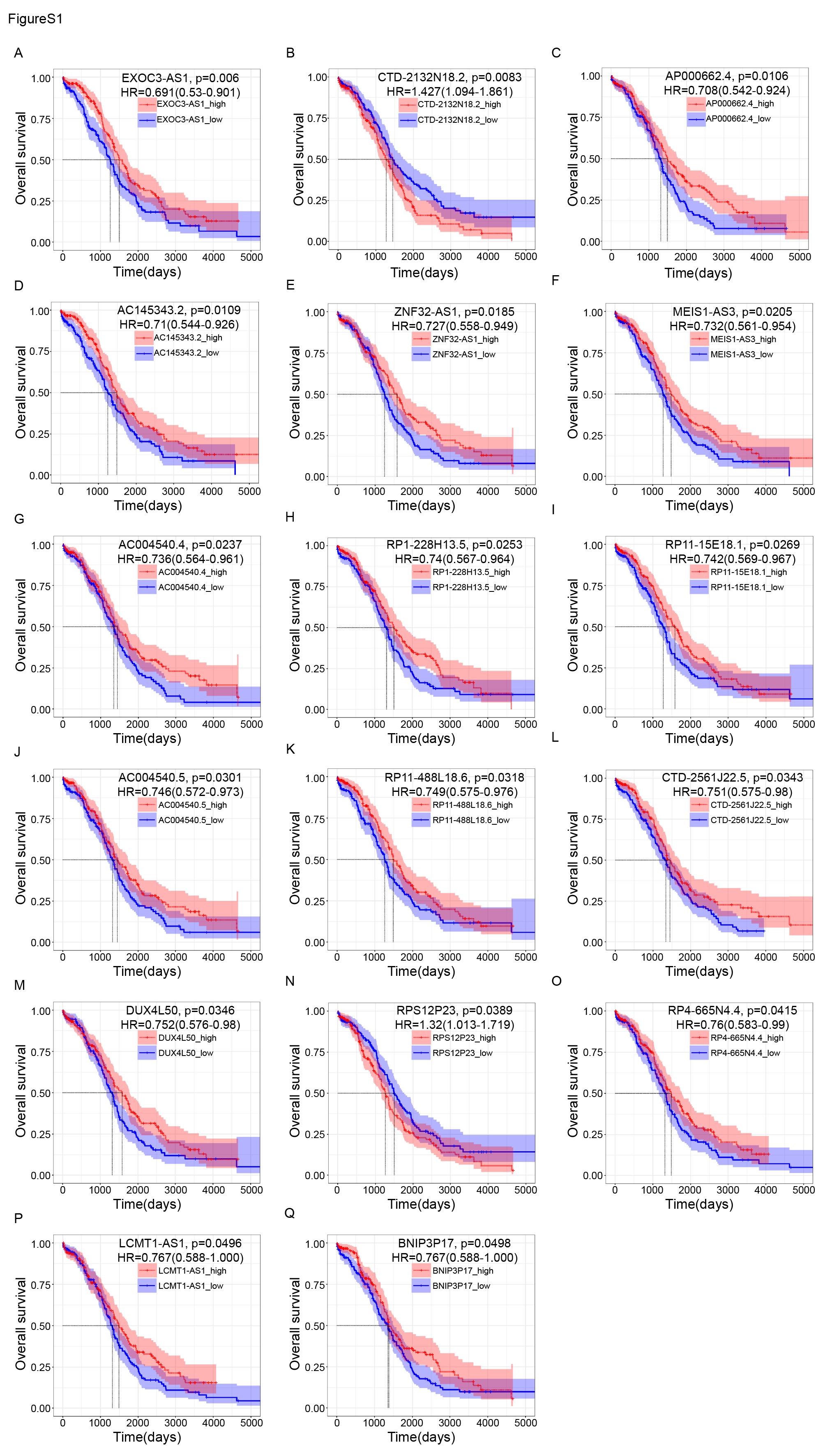

Supplement: Supplementary file 9 — Additional file 9: Figure S1. Survival analysis of lncRNAs in intersection. (A-Q) Kaplan-Meier survival curves of overall survival times between the high-expression and low-expression group of EXOC3-AS1 (A), CTD-2132N18.2 (B), AP000662.4 (C), AC145343.2 (D), ZNF32-AS1 (E), MEIS1-AS3 (F), AC004540.4 (G), RP1-228H13.5 (H), RP11-15E18.1 (I), AC004540.5 (J), RP11-488L18.6 (K), CTD-2561J22.5 (L), DUX4L50 (M), RPS12P23 (N), RP4-665N4.4 (O), LCMT1-AS1 (P), BNIP3P17 (Q). [file 13048_2023_1260_MOESM9_ESM.tif]

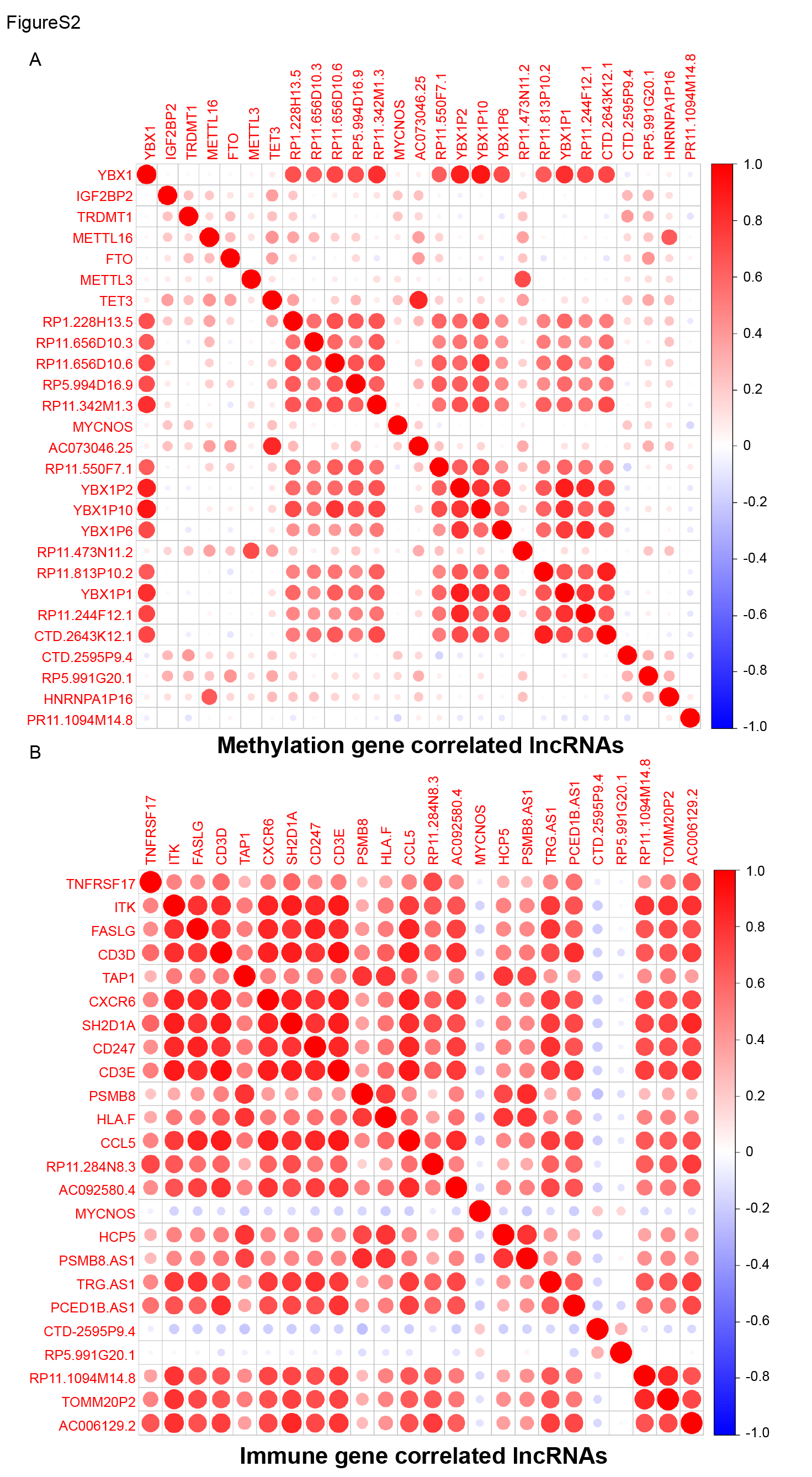

Supplement: Supplementary file 10 — Additional file 10: Figure S2. lncRNAs strongly correlated with methylation genes and immune genes. (A) The top 20 lncRNAs strongly correlated with methylation genes. (B) The top 20 lncRNAs strongly correlated with immune genes. The process used the criteria of |Pearson R| >0.35 and p <0.05. [file 13048_2023_1260_MOESM10_ESM.tif]

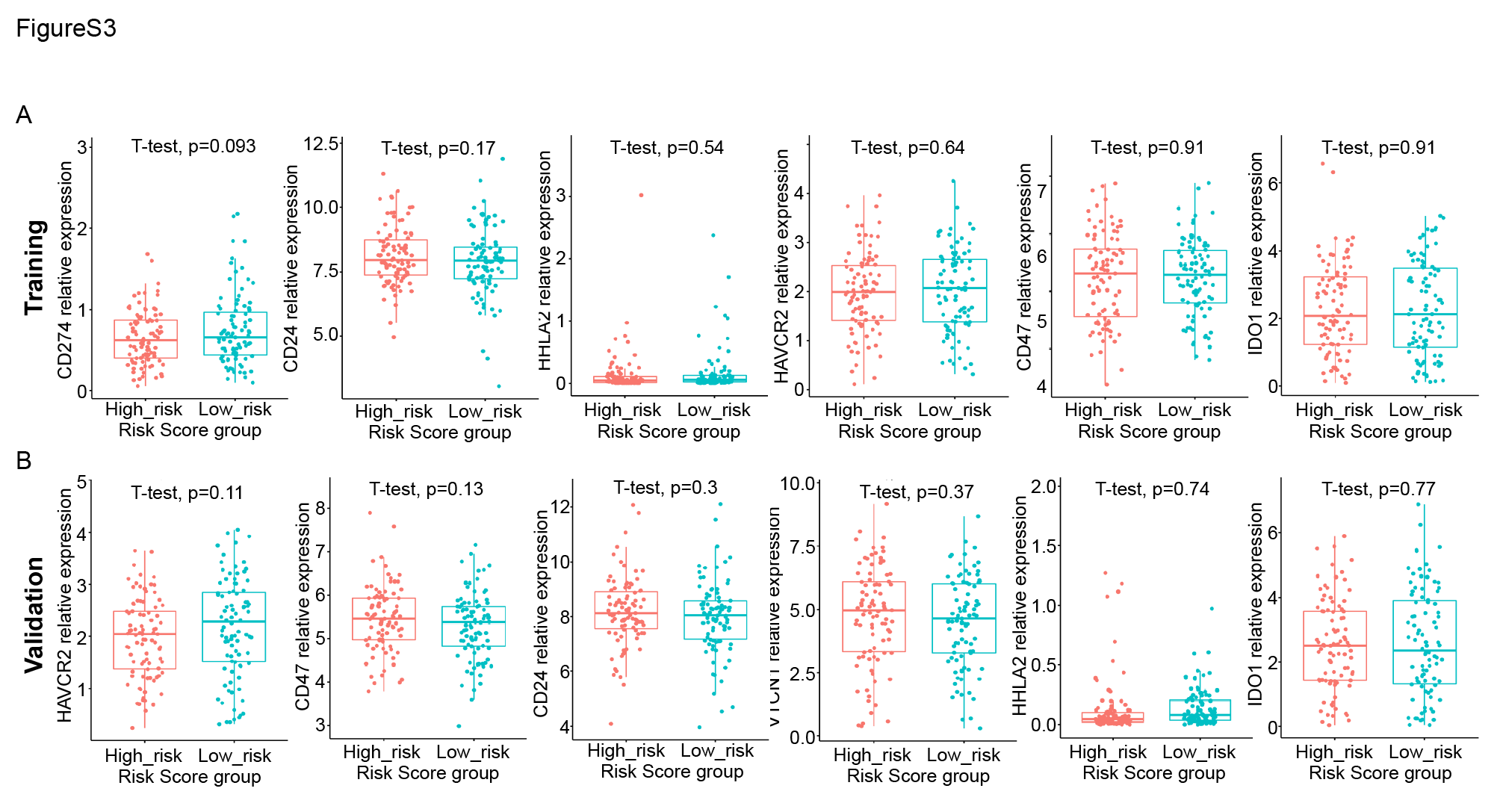

Supplement: Supplementary file 11 — Additional file 11: Figure S3. Correlation histogram of immune checkpoints. Correlation histogram of immune checkpoints related to riskscore in training set (A) and validation set (B). [file 13048_2023_1260_MOESM11_ESM.tif]
